# Neuroarchitecture of the *Drosophila* central complex: A catalog of nodulus and asymmetrical body neurons and a revision of the protocerebral bridge catalog

**DOI:** 10.1002/cne.24512

**Published:** 2018-10-18

**Authors:** Tanya Wolff, Gerald M. Rubin

**Affiliations:** ^1^ Janelia Research Campus, Howard Hughes Medical Institute Ashburn Virginia

**Keywords:** AB_1549585, AB_1625981, AB_2314866, AB_915420, asymmetrical body, central complex, *Drosophila* brain, GAL4, MCFO, nodulus, protocerebral bridge

## Abstract

The central complex, a set of neuropils in the center of the insect brain, plays a crucial role in spatial aspects of sensory integration and motor control. Stereotyped neurons interconnect these neuropils with one another and with accessory structures. We screened over 5,000 *Drosophila melanogaster* GAL4 lines for expression in two neuropils, the noduli (NO) of the central complex and the asymmetrical body (AB), and used multicolor stochastic labeling to analyze the morphology, polarity, and organization of individual cells in a subset of the GAL4 lines that showed expression in these neuropils. We identified nine NO and three AB cell types and describe them here. The morphology of the NO neurons suggests that they receive input primarily in the lateral accessory lobe and send output to each of the six paired noduli. We demonstrate that the AB is a bilateral structure which exhibits asymmetry in size between the left and right bodies. We show that the AB neurons directly connect the AB to the central complex and accessory neuropils, that they target both the left and right ABs, and that one cell type preferentially innervates the right AB. We propose that the AB be considered a central complex neuropil in *Drosophila*. Finally, we present highly restricted GAL4 lines for most identified protocerebral bridge, NO, and AB cell types. These lines, generated using the split‐GAL4 method, will facilitate anatomical studies, behavioral assays, and physiological experiments.

## 1 INTRODUCTION

Located at the center of the insect brain, the central complex is a set of highly interconnected neuropils that processes complex, multisensory information from the environment, integrates it with information about the insect's internal state and past experiences, and guides motor outputs that drive appropriate behavioral responses. A comprehensive review of the functional roles of the central complex in diverse insects can be found in Pfeiffer and Homberg (2014).

One of the most studied roles of the insect central complex is the integration of sensory information, predominantly from visual input. The output of this sensory processing encompasses diverse motor and behavioral responses. In this capacity, the central complex regulates locomotor behaviors such as handedness, turn direction, initiation and termination of walking (Buchanan, Kain, & de Bivort, 2015; Guo & Ritzmann, 2013; Martin, Guo, Mu, Harley, & Ritzmann, 2015; Martin, Raabe, & Heisenberg, 1999; Poeck, Triphan, Neuser, & Strauss, 2008; Ritzmann, Ridgel, & Pollack, 2008; Seelig & Jayaraman, 2013); flight (Ilius, Wolf, & Heisenberg, 1994); courtship (Sakai & Kitamoto, 2006); sleep (Donlea, Pimentel, & Miesenbock, 2014; Liu, Liu, Tabuchi, & Wu, 2016); hunger (Park et al., 2016); and gravitaxis (Baker, Beckingham, & Armstrong, 2007). The central complex is thought to play a key role in migration, navigation, and orientation using input such as celestial cues (el Jundi et al., 2015; Kakaria & de Bivort, 2017; Seelig & Jayaraman, 2015; Kuntz, Poeck, & Strauss, 2017) and displays responses to looming stimuli suggestive of an involvement in generating escape responses in the locust and fly (Rosner & Homberg, 2013; Weir, Schnell, & Dickinson, 2014). The central complex has been suggested to contain a ring attractor network (Kim, Rouault, Druckmann, & Jayaraman, 2017; Seelig & Jayaraman, 2015) that maintains a representation of the fly's heading direction that may be useful for navigation and orientation in visual conditions as well as in darkness (Green et al., 2017; Seelig & Jayaraman, 2015; Turner‐Evans et al., 2017). The central complex is also involved in the formation and recall of short‐ and long‐term visual memories (Liu et al., 2006; Neuser, Triphan, Mronz, Poeck, & Strauss, 2008; Ofstad, Zuker, & Reiser, 2011), in the processing of olfactory (Heisenberg, Borst, Wagner, & Byers, 1985) and gustatory inputs (Bouhouche, Vaysse, & Corbiere, 1993) and in maintaining information about the fly's satiety state (Dus, Min, Keene, Lee, & Suh, 2011).

**Figure 1:**
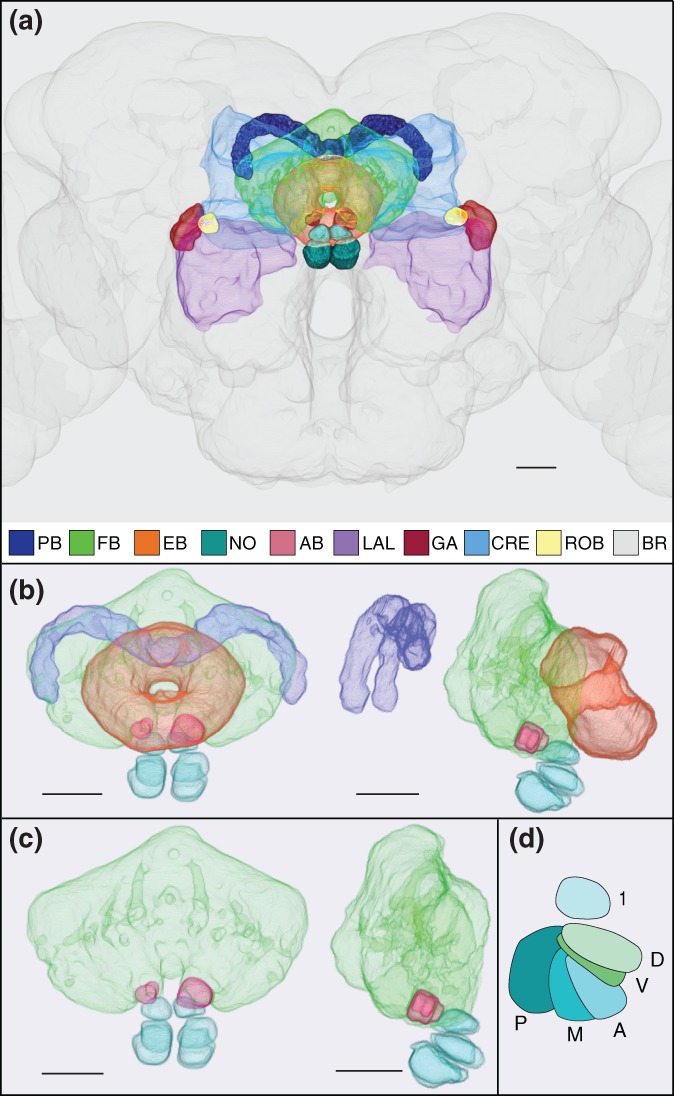
The central complex and accessory neuropils. (a) Neuropils of the central complex and accessory regions. Frontal view as seen from the anterior. Neuropil masks were created in FluoRender (https://bmcbioinformatics.biomedcentral.com/articles/10.1186/s12859-017-1694-9 or (Wan, Otsuna, Chien, & Hansen, 2009, 2012) and were aligned in the JRC 2013 brain. The color coding is as follows. PB: dark blue; FB = green; EB = orange; NO = teal; AB = rose (dorsal to NO); LAL = lilac; GA = burgundy (“shoulders” of LAL); crepine (CRE) = light blue; round body (ROB) = yellow; brain = gray. Scale bar = 20 μ. (b) Frontal (left) and sagittal (right) views of central complex neuropils. Anterior is to the right in the sagittal view. PB = purple; FB = green, EB = orange; NO = cyan; AB = rose. Scale bar = 20 μ. (c) The same images as shown in (b), with the PB and EB removed to better visualize the location of the AB (rose). Scale bar = 20 μ. (d) Sagittal view of one set of noduli. The dorsal nodulus, NO_1_ (1), is not horizontally segmented. The medial nodulus, NO_2_, has two subcompartments, dorsal (D) and ventral (V). The ventral nodulus, NO_3_, has three subcompartments, anterior (A), medial (M), and posterior (P)

Understanding the core principles of operation of the central complex has been greatly enabled by the dissection of behavior at a single neuron level and the neuron‐by‐neuron assembly of circuits. A comprehensive anatomical atlas and genetic lines that enable manipulation of individual cell types are invaluable tools for this strategy. In this study, we describe the neuronal composition of the NO and the AB, neither of which has been extensively studied in *Drosophila*. An understanding of the function of the noduli (Figure 1a–d) in behavior lags far behind that of the other central complex structures: the protocerebral bridge (PB), fan‐shaped body (FB), and ellipsoid body (EB; Figure 1a,b; see Table 1 for abbreviations). The only documented roles for the NO in *Drosophila* are in the time course of walking activity (Strauss & Heisenberg, 1993) and in influencing handedness during locomotion (Buchanan et al., 2015). The locust neurons that connect the PB, EB, and NO and the PB, FB, and NO are sensitive to polarized light (Heinze & Homberg, 2009). Most recently, recordings from optic‐flow‐sensitive neurons that connect the LAL to the NO and other neurons that link the NO to the FB in the bee have demonstrated the NO are involved in path integration (Stone et al., 2017). Finally, the fact that this structure appears to be present only in the subclass of winged insects has led to the speculation that the noduli may regulate flight (Pfeiffer & Homberg, 2014).

**Table 1:** Abbreviations

PB	protocerebral bridge
FB	fan‐shaped body
EB	ellipsoid body
EBc	canal (EB domain)
EBt	tile (EB domain)
NO	nodulus
NO_1_	nodulus 1, dorsal nodulus
NO_2_	nodulus 2, medial nodulus
NO_2_D	dorsal compartment of NO_2_
NO_2_V	ventral compartment of NO_2_
NO_3_	nodulus 3, ventral nodulus
NO_3_A	anterior compartment of NO_3_
NO_3_M	medial compartment of NO_3_
NO_3_P	posterior compartment of NO_3_
AB	asymmetrical body
LAL	lateral accessory lobe
GA	gall
GA‐t	gall tip
CRE	crepine
ROB	round body
SMP	superior medial protocerebrum
SLP	superior lateral protocerebrum
SCL	superior clamp
VES	vest
WED	wedge
G	glomerulus of PB
G#	subset of glomeruli designated by number/s
ℓ	layer (of FB)
s	spines/spiny
b	boutons
D	dorsal
V	ventral
P	posterior
i	ipsilateral
c	contralateral
SS	stable split
MCFO	multicolor flip‐out

Structural conservation of the central complex across insect species is strong, but not absolute. The discussion that follows focuses on the anatomy of the *Drosophila* neuropils (Figure 1), unless otherwise noted. The PB, FB, EB, and NO are midline structures and exhibit a stratified organization (Heinze & Homberg, 2008; Homberg, 2008; Strausfeld, 1999; Williams, 1975). The PB is a handlebar‐shaped structure in the posterior dorsal brain (Figure 1a,b). The EB is shaped like a torus and is tilted on its dorsoventral axis such that its ventral border defines the anterior margin of the central complex (Figure 1a,b). The FB lies between the PB and EB and represents the largest of the four central complex structures (Figure 1a–c). The bilateral noduli, historically called ventral tubercles (Hertweck, 1931), are the most ventral neuropil of the central complex and are nestled beneath the FB (Figure 1a–c). There are three pairs of noduli neatly stacked on top of one another from dorsal to ventral; each pair is bisected by the midline (Figure 1a–c; one set is shown in panel d). The dorsal nodulus (NO_1_) displays some hint of a transverse division (see Wolff, Iyer, & Rubin, 2015) whereas the medial (NO_2_) and ventral (NO_3_) noduli exhibit longitudinal segmentation. NO_2_ is divided into dorsal and ventral subdomains (NO_2_D and NO_2_V; D and V, respectively, in Figure 1d) and NO_3_ has three subdomains (NO_3_A, NO_3_M, and NO_3_P; A, M, and P, respectively, in Figure 1d; see Wolff et al., 2015 for details).

The neuropils considered to be components of the central complex have evolved over time. Power's 1943 description of the central complex includes the FB, EB, and “ventral tubercles” (Power, 1943), or NO. By the mid‐1970s, the modern view of the central complex had emerged: Williams included the PB within the locust central complex, alongside the FB, EB and NO (Williams, 1975).

The asymmetrical body (AB) is a relatively inconspicuous structure located at the midline, adjacent to the ventral FB. It was first described in the fly as a round, almost exclusively right‐hemisphere structure (Pascual, Huang, Neveu, & Preat, 2004). The AB was observed in both hemispheres in just 7.6% of 2,250 brains immunolabeled with an antibody against the Fasciclin II (Fas II) protein, which is expressed in this structure. Flies with bilateral ABs were reported to have disrupted long‐term memory, leading to the suggestion that asymmetry of this structure is important for long‐term, but not short‐term, memory (Pascual et al., 2004). Although thousands of GAL4 lines that drive expression in small subsets of neurons in the larval and adult fly brains have been examined (Jenett et al., 2012), the AB is the only reported instance of an asymmetric structure in the adult fly brain.

Elements with likely homology to the *Drosophila* AB have also been described in the grey flesh fly *Neobellieria bullata* and the blowfly *Calliphora erythrocephala* (Phillips‐Portillo & Strausfeld, 2012). In both species, these bodies occur bilaterally, and one of the two is consistently smaller and less densely innervated than the other. In addition, the smaller of the two appears fragmented.

Jenett et al. (2012) identified five GAL4 lines that show asymmetric innervation of the AB. Their analysis revealed lines ranging from a strong right hemisphere bias in innervation to those with asymmetric but bilateral expression, with more conspicuous expression in the right AB. This study builds on Jenett et al. (2012) by providing a systematic characterization of the neurons that target the AB, and leads us to propose that the AB be added as the fifth neuropil of the *Drosophila* central complex.

In this work, we present a characterization of cell types of the NO and AB, including morphology, presumed polarity, and population size. We also generated and characterized a set of split‐GAL4 lines for NO and AB cell types, reagents that will greatly facilitate functional studies. These lines are presented here in Table 2.

**Table 2:** Stable split GAL4 lines and cell number per cell type

Cell type	Cell#/hem	1 ° SS line	2 ° SS line	2 ° SS line	2 ° SS line	2 ° SS line
**PB neurons**						
PB_G2‐9_.s‐FBℓ1.b‐NO_3_P.b/PB_G2‐9_.s‐FBℓ1.b‐NO_3_M.b	32	**SS52244**	SS00191	SS00425		
PB_G2‐9_.s‐FBℓ2.b‐NO_3_A.b	12	**SS02255**	SS00081	SS00043	SS00190	
PB_G2‐9_.s‐FBℓ1.b‐NO_3_P.b&NO_3_M.b&NO_3_A.b^a^		**SS52245**	SS02303	SS00154	SS00159	
PB_G2‐9_.s‐FBℓ3.b‐NO_2_D.b	18	**SS00078**	SS52669	SS52338		
PB_G2‐9_.s‐FBℓ3.b‐NO_2_V.b	9–11	**SS52577**	SS52628	SS53161	SS53191	
PB_G2‐9_.s‐EBt.b‐NO_1_.b	9–10	**SS54295**	SS52285^b^	SS04912^c^	SS02233	
PB_G1‐9_.s‐EBt.b‐D/V GA.b	9	**SS27853**	**SS02191**	SS53233		
PB_G1‐9_.s‐EBc.b‐D/V GA.b	9	**SS02195**	SS53236	SS56252		
PB_G1‐8_.b‐EBw.s‐D/V GA.b	18–21	**SS00090**	**SS50574**	**SS00408**	**SS00098**	
PB_G9_.b‐EB.P.s‐GA‐t.b	1–2	**SS02254**	SS50594	SS50576		
PB.s‐FBℓ6.b.ℓ3.s‐V GA‐s.b	17–22	**SS52590**	SS53175	SS52547		
PB_G1‐8_.s‐FBℓ3,4,5.s.b‐ROB.b	14–17	**SS54549**	SS02270	SS02293		
PB_G17_.s‐FBℓ2.s‐LAL.b‐cre.b	7	**SS02239**	SS02215	SS53185		
PB.b‐LAL.s‐PS.s	1	**SS52578**	SS52604	SS52684		
PB_G6‐8_.s_G9_.b	2	**SS00117**	SS52272	SS52347		
PB18.s‐GxΔ7Gy.b/PB18.s‐9i1i8c.b	9–10	**SS52266**	**SS52257**	**SS52313**		
PB_G1/2‐9_.b‐SPSi.s	2	**SS52267**	SS52265	SS56247		
PB_G2‐9_.b‐IB.s.SPS.s	8–12	**SS04778**	**SS25983**	SS03950		
PB_G1‐9_.s‐EBt.b‐D/V GA.b&PB_G1‐9_.s‐EBc.b‐D/V GA.b		SS02198	SS04773			
**NO neurons**						
LAL.s‐GAi.s‐NO_1_i.b	2	**SS46517**	**SS46524**	SS46512	SS46515	
LAL.s‐NO_2_i.b	2	**SS47398**	SS47399	SS47378	SS47405	SS47354
LAL.s‐NO_3_Ai.b	1	**SS47432**	SS47436	SS47449	SS47406^d^	
LAL.s‐CREi.s‐NO_3_P/Mi.b	1	**SS47384**	**SS47392**	SS47467	SS47356	SS47410
LAL.s‐CREc.s‐NO_3_Pc.b	1	**SS46525**	SS46528	SS46507	SS46523	
**AB neurons**						
SLP‐AB	3	**SS50464**	**SS50498**	**SS50502**	**SS50510**	
AB‐FBℓ8	13–19	**SS02718**	SS04444	SS50447	SS50487	
SLP‐AB‐FBℓ8	2	**SS50420**	**SS50417**	**SS50477**	**SS50517**	

Cell type(s) and number of cells per hemisphere are provided for each line. One primary (best) and several good lines/cell type are provided. Some 2 ° lines are equally as good as the 1 ° lines; these are indicated in bold.

a PB_G2‐9_.s‐FBℓ1.b‐NO_3_P.b&NO_3_M.b&NO_3_A.b contains PB_G2‐9_.s‐FBℓ1.b‐NO_3_P.b, PB_G2‐9_.s‐FBℓ1.b‐NO_3_M.b, PB_G2‐9_.s‐FBℓ2.b‐NO_3_A.b.

b Also has weak expression in PB_G2‐9_.s‐FBℓ3.b‐NO_2_V.b.

c This line targets P‐EN2 cells (Dan Turner‐Evans, personal communication; see Green et al., 2017, for description of cell type). The identities of the P‐EN cells in the first two lines have not been characterized. Also has weak expression in PB_G2‐9_.s‐FBℓ3.b‐NO_2_V.b.

d This line is stochastic—expression may occur in only one hemisphere.

In addition, since publication of our description of the neurons that arborize in the PB (Wolff et al., 2015), we have gained several new insights into the PB neurons. These include (a) one new PB neuron family has been identified; (b) a neuron identified by Lin et al. (2013) has since been found in the GAL4 collection and is characterized here using the multicolor flip‐out technique (MCFO; Nern, Pfeiffer, & Rubin, 2015); and (c) we generated and characterized a set of split‐GAL4 lines for PB cell types; these are presented in the Results section in Table 2.

## 2 MATERIALS AND METHODS

### 2.1 Split‐GAL4 methodology and lines

In the split‐GAL4 method, the DNA‐binding domain (DBD) and activation domain (AD) of the GAL4 transcription factor are separately linked to complementary leucine zippers and placed under the control of different gene enhancers to create “hemidriver lines” (Luan, Peabody, Vinson, & White, 2006; Pfeiffer et al., 2010). Each enhancer drives gene expression in an empirically determined population of neurons. In cells in which both enhancers are active, both the AD and DBD are expressed and can dimerize via their leucine zippers to reconstitute transcriptionally active GAL4 protein. The reconstituted GAL4 protein binds to the upstream activating sequence (UAS) and activates expression of the UAS‐transgenes. In this case, the UAS‐transgenes used are “reporters” (e.g., GFP) that visually identify those cells that express GAL4.

Adult brain expression patterns of approximately 8,200 GAL4 lines, 7,000 from the Janelia collection (Jenett et al., 2012; Pfeiffer et al., 2008) and close to 1,200 from the Vienna collection (Tirian & Dickson, 2017), were screened for enhancer fragments that drive expression in the central complex. Hemidriver lines in which these enhancer fragments drive expression of either the GAL4 activation domain (AD; p65ADZp) or DNA‐binding domain (DBD; ZpGAL4DBD) were subsequently selected from a collection of transgenic lines (Dionne, Hibbard, Cavallaro, Kao, & Rubin, 2018; Tirian & Dickson, 2017). AD and DBD hemidriver lines judged to drive expression in common cell types were crossed to one another. The progeny of these crosses was screened to identify combinations in which the GAL4 AD and DBD domains are both expressed in the desired cell type and can combine to produce functional GAL4 protein. Stable genetic stocks containing both AD and DBD hemidriver constructs (stable split, or SS, lines; Table 2) were generated for the most specific lines for each cell type and these split‐GAL4 lines were analyzed using the multicolor flip‐out method (MCFO; see below) and polarity markers. Details on the methodology of this approach can be found in Dionne et al. (2018). The split‐GAL4 lines generated for each cell type are shown in Table 2 and the hemidriver constructs used to make each line are listed in Table 3.

**Table 3:** Parental AD and DBD Hemidriver lines

**SS00078** GMR_16D01_XA_21‐x‐GMR_15E01_XD_01	**SS52577** BJD_109C02_BB_21‐x‐BJD_126C06_AV_01
SS52669 GMR_68A10_BB_21‐x‐GMR_75H10_XD_01	SS52628 GMR_22G07_BB_57‐x‐BJD_109C02_AV_01
SS52338 GMR_75H10_XA_21‐x‐GMR_83D12_AV_01	SS53161 BJD_109C02_BB_21‐x‐BJD_105F11_AV_01
	SS53191 GMR_22G07_BB_37‐x‐BJD_105F11_AV_01
**SS00090** GMR_19G02_XA_21‐x‐GMR_15C03_XD_01	**SS52578** BJD_110F01_BB_21‐x‐BJD_101G02_AV_01
**SS50574** BJD_113B09_BB_21‐x‐BJD_114B09_AV_01	SS52604 BJD_126C06_BB_21‐x‐BJD_105C09_AV_01
**SS00408** GMR_27F02_XA_21‐x‐GMR_19G02_XD_01	SS52684 GMR_89G08_BB_21‐x‐BJD_113C04_AV_01
**SS00098** GMR_19G02_XA_21‐x‐GMR_93G12_XD_01	
**SS00117** GMR_24A02_XA_21‐x‐GMR_18G01_XD_01	**SS52590** BJD_113B09_BB_21‐x‐BJD_100A02_AV_01
SS52272 BJD_123G10_BB_21‐x‐GMR_24A02_AV_01	SS53175 BJD_121H10_BB_21‐x‐BJD_100A02_AV_01
SS52347 GMR_92B02_XA_21‐x‐GMR_48H01_AV_01	SS52547 BJD_100A02_BB_21‐x‐BJD_119A12_AV_01
**SS02195** BJD_100A02_BB_21‐x‐GMR_47A08_XD_01	**SS54295** BJD_126F12_BB_21‐x‐BJD_107H03_AV_01
SS53236 GMR_84H05_BB_04‐x‐GMR_51E05_AV_01	SS52285 GMR_13D05_XA_21‐x‐GMR_48A11_AV_01
SS56252 GMR_38B06_XA_21‐x‐BJD_115C10_AV_01	SS04912 GMR_41G11_BB_21‐x‐GMR_72G09_AV_01
	SS02233 BJD_120E09_BB_21‐x‐BJD_125G02_AV_01
SS02198 BJD_100A02_BB_21‐x‐GMR_89F06_AV_01	**SS54549** GMR_38B06_XA_21‐x‐BJD_126G08_AV_01
SS04773 GMR_28G07_BB_21‐x‐BJD_100A02_AV_01	SS02270 GMR_30G06_XA_21‐x‐GMR_27A10_XD_01
	SS02293 GMR_41G11_XA_21‐x‐BJD_106E02_AV_01
**SS02239** GMR_15C05_XA_21‐x‐GMR_15A05_XD_01	**SS46517** BJD_115B11_BB_21‐x‐BJD_120E12_AV_01
SS02215 BJD_112D01_BB_21‐x‐GMR_13D09_XD_01	**SS46524** BJD_116C06_BB_21‐x‐BJD_120E12_AV_01
SS53185 GMR_11B11_BB_21‐x‐BJD_122C03_AV_01	SS46512 BJD_111A03_BB_21‐x‐BJD_120E12_AV_01
	SS46515 BJD_111A03_BB_21‐x‐GMR_76E11_AV_01
**SS02254** GMR_15E01_XA_21‐x‐BJD_103H04_AV_01	**SS46525** BJD_116C06_BB_21‐x‐BJD_125B02_AV_01
SS50594 GMR_93G12_XA_21‐x‐GMR_65H10_AV_01	SS46528 BJD_118C01_BB_21‐x‐BJD_105D12_AV_01
SS50576 BJD_113B09_BB_21‐x‐GMR_65H10_AV_01	SS46507 BJD_105D12_BB_21‐x‐BJD_101D06_AV_01
	SS46523 BJD_116C06_BB_21‐x‐BJD_118C01_AV_01
**SS02255** GMR_16D01_XA_21‐x‐BJD_103H04_AV_01	**SS47384** BJD_111G03_BB_21‐x‐BJD_115C01_AV_01
SS00081 GMR_16D01_XA_21‐x‐GMR_21H11_XD_01	**SS47392** BJD_112D04_BB_21‐x‐BJD_115C01_AV_01
SS00043 GMR_65B12_XA_21‐x‐GMR_30E10_XD_01	SS47467 GMR_48H02_BB_21‐x‐BJD_101F10_AV_01
SS00190 GMR_38G07_XA_21‐x‐GMR_30E10_XD_01	SS47356 BJD_101F10_BB_21‐x‐BJD_105B02_AV_01
	SS47410 BJD_115C01_BB_21‐x‐BJD_112D04_AV_01
**SS04778** GMR_47G08_BB_21‐x‐BJD_109G05_AV_01	**SS47398** BJD_113C06_BB_21‐x‐BJD_100H07_AV_01
**SS25983** GMR_47G08_BB_21‐x‐GMR_26F04_AV_01	SS47399 BJD_113C06_BB_21‐x‐BJD_111C05_AV_01
SS03950 GMR_44A02_BB_21‐x‐GMR_13G10_XD_01	SS47378 BJD_111C05_BB_21‐x‐BJD_104A08_AV_01
	SS47405 BJD_113C06_BB_21‐x‐GMR_73E12_AV_01
	SS47354 BJD_100H07_BB_21‐x‐BJD_104A08_AV_01
**SS27853** GMR_33A12_BB_37‐x‐BJD_108B12_AV_01	**SS47432** BJD_128B05_BB_21‐x‐BJD_110C07_AV_01
**SS02191** BJD_100A02_BB_21‐x‐BJD_108B12_AV_01	SS47436 GMR_12G04_XA_21‐x‐BJD_128B05_AV_01
SS53233 GMR_84H05_BB_04‐x‐BJD_100A02_AV_01	SS47449 GMR_38D03_BB_21‐x‐BJD_110C07_AV_01
	SS47406 BJD_115B11_BB_21‐x‐BJD_110C07_AV_01
**SS52244** BJD_111B06_BB_21‐x‐GMR_48A11_AV_01	**SS02718** BJD_103D04_BB_21‐x‐GMR_83D12_AV_01
SS00191 GMR_38G07_XA_21‐x‐GMR_37H01_XD_01	SS04444 GMR_21A07_BB_21‐x‐BJD_110B06_AV_01
SS00425 GMR_65B12_XA_21‐x‐GMR_85H06_XD_01	SS50447 BJD_119G09_BB_21‐x‐BJD_100D11_AV_01
	SS50487 GMR_25B07_BB_21‐x‐BJD_120D05_AV_01
**SS52245** BJD_111B06_BB_21‐x‐GMR_83D12_AV_01	**SS50420** BJD_107B07_BB_21‐x‐GMR_48H02_AV_01
SS02303 GMR_65B12_XA_21‐x‐GMR_15E12_AV_01	**SS50417** BJD_107B07_BB_21‐x‐BJD_100A07_AV_01
SS00154 GMR_37H01_XA_21‐x‐GMR_40A01_XD_01	**SS50477** BJD_123E11_BB_21‐x‐BJD_107B07_AV_01
SS00159 GMR_37H01_XA_21‐x‐GMR_79A12_XD_01	**SS50517** GMR_48H02_BB_21‐x‐BJD_123E11_AV_01
**SS52266** BJD_122F02_BB_21‐x‐GMR_38G02_AV_01	**SS50464** BJD_122E10_BB_21‐x‐BJD_111D08_AV_01
**SS52257** BJD_121E12_BB_21‐x‐GMR_24A02_AV_01	**SS50498** GMR_26C06_XA_21‐x‐BJD_122E10_AV_01
**SS52313** GMR_45G06_XA_21‐x‐GMR_27G01_AV_01	**SS50502** GMR_26C06_XA_21‐x‐GMR_72A10_AV_01
	**SS50510** GMR_42C09_XA_21‐x‐BJD_122E10_AV_01
**SS52267** BJD_122F02_BB_21‐x‐GMR_72C10_AV_01	
SS52265 BJD_122F02_BB_21‐x‐GMR_37H01_AV_01	
SS56247 BJD_122F02_BB_21‐x‐BJD_122C09_AV_01	

The lines shown in Table 3 correspond to those shown in Table 2. Lines are arranged numerically by primary SS line number and in ascending order. They are further arranged by neuropil region, starting with the PB, followed by the NO and last by the AB. Each text box in Table 3 includes all lines for a given cell type that are presented in Table 2. SS lines indicated in bold are the same lines shown in bold in Table 2 and identify the best lines for each cell type.

### 2.2 Using the MCFO method to screen for and characterize cell types

Of the 8,900 GAL4 and split‐GAL4 lines screened for expression in the central complex, as described above, approximately 1,200 were selected for analysis by MCFO. The MCFO technique stochastically labels a small number of cells in various colors (Nern et al., 2015), making it possible to analyze the morphology of individual cells at high resolution using confocal microscopy. The MCFO strategy was used to identify and characterize the NO and AB cell types described here. Briefly, the fly stocks used for MCFO contain three UAS reporter constructs (UAS‐FRT‐STOP‐FRT‐epitope tag), each with a different epitope tag (Flag, VK5, or HA). Flp recombinase‐mediated excision (Struhl & Basler, 1993) stochastically removes the STOP cassette in a small number of cells, enabling expression of one to three epitopes. The epitope tags are subsequently labeled with appropriate antibodies (Table 4). The combination of tags expressed dictates each labeled neuron's color.

**Table 4:** Primary antibodies used in this study

Antibody	Immunogen	Source	Dilution
Anti‐Bruchpilot	Amino acids 1,105–1,740 of *Drosophila* Bruchpilot C‐terminus	DSHB, mouse, monoclonal, nc82, RRID: AB_2314866	1:30
Anti‐HA	Influenza HA epitope YPYDVPDYA	Cell Signaling Technology, 3724S, rabbit, monoclonal, RRID: AB_1549585	1:300
Anti‐FLAG	N‐terminal DYKDDDDK‐tagged ECD of mouse Langerin	Novus Biologicals, NBP1–06712, rat, monoclonal, RRID:AB_1625981	1:200
Anti‐V5	Paramyxovirus SV5	AbD Serotec, MCA 1360D549, mouse, monoclonal, RRID: AB_915420	1:500

In addition, searches for both NO and AB neurons were performed against a database of “MCFO brains” (adult brains prepared using the MCFO technique) representing 2,200 GAL4 lines. (These 2,200 lines represent a subset of the 8,900 GAL4 lines described earlier.) In these “color depth mask searches,” a binarized image stack of the neuron to be searched was projected into two‐dimensional space and colored by its position in the *z*‐axis (the “mask”). This mask was used to search a database of aligned neurons for pixel overlap using a comparison algorithm described in https://www.biorxiv.org/content/early/2018/05/09/318006.

The following fly stock was used for MCFO: [pBPhsFlp2::PEST in attP3;; pJFRC201‐10XUAS‐FRT > STOP>FRT‐myr::smGFP‐HA in VK00005,pJFRC240‐10XUAS‐FRT > STOP>FRT‐myr::smGFP‐V5‐THS‐10XUAS‐FRT > STOP>FRT‐myr::smGFP‐FLAG in su[Hw]attP1/TM3, Sb]. GAL4 and split‐GAL4 stocks are noted throughout the text. Details concerning constructs and methods for the MCFO technique are described in Nern et al. (2015). Briefly, crosses were set up (day 1) and maintained at 21°C. Flies eclosed on day 14–15 and were heat shocked on day 16 (1–2 days old) by placing the vials containing the flies in a 37°C water bath for 15 min. Female flies were used almost exclusively in this study (some male flies were analyzed for the AB portion of the study) and were dissected 5–9 days following heat shock. Tissue was processed as outlined below.

### 2.3 Split‐GAL4 expression patterns

**Figure 2:**
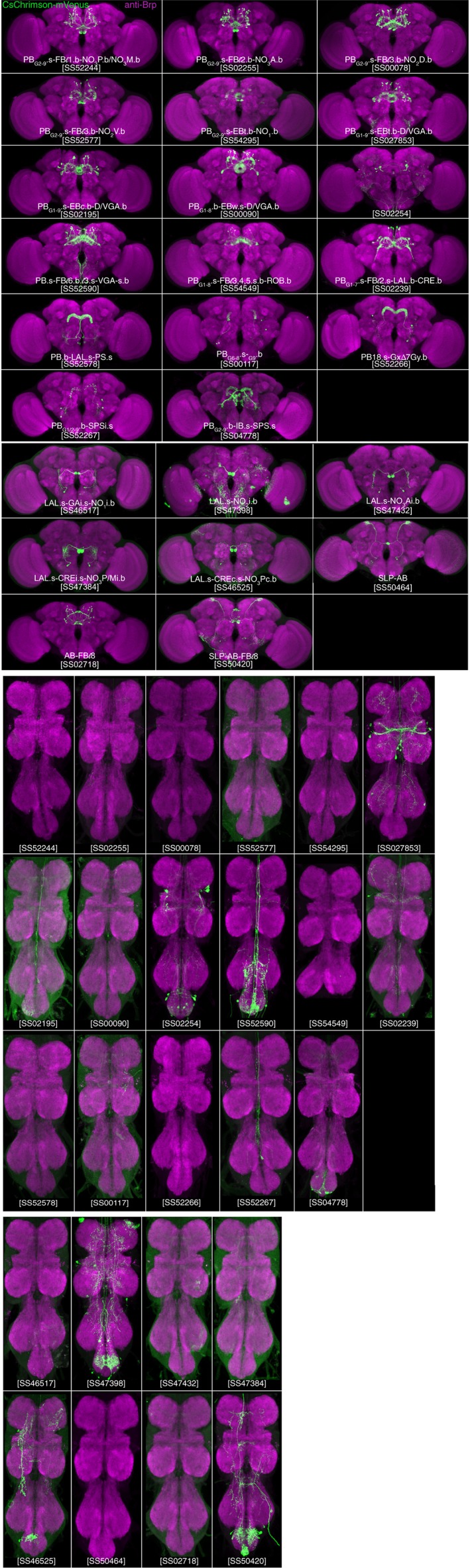
Expression patterns of PB, NO, and AB split‐GAL4 lines in brain and VNC. Expression of 20xUAS‐CsChrimson‐mVenus (insertion in attP18; labeled with anti‐GFP antibody, green) driven by split‐GAL4 lines indicated in panels. Neuropil was visualized using anti‐Brp, shown in magenta. The sparsest lines for each cell type are shown. Confocal stacks for these and additional lines noted in Table 2 can be viewed and lines can be ordered at http://www.janelia.org/split-GAL4. This website will be updated with additional clean lines as they become available. Scale bar = 100 μ

The expression patterns of split‐GAL4 lines are visualized in Figure 2 using 20XUAS‐CsChrimson‐mVenus in *attP18* (Klapoetke et al., 2014) as the neuronal marker and anti‐BRP to label the neuropil. Expression patterns of additional lines are shown at http://www.janelia.org/split-GAL4 and confocal stacks corresponding to all split‐GAL4 lines described here can be downloaded from that site. Instructions for requesting split‐GAL4 lines can also be found at this site. To supplement the anatomy‐based assignments of neuronal polarity, split‐GAL4 lines were used to drive the expression of two reporter constructs, one targeted to membranes (pJFRC225‐5XUAS‐IVS‐myr::smGFP‐FLAG in VK00005); (Nern et al., 2015; Viswanathan et al., 2015) and a second that localizes to synaptic vesicles in presynapses (pJFRC51‐3XUAS‐IVS‐Syt::smGFP‐HA in su(Hw)attP1). Synaptotagmin (Syt) is a presynaptic protein (Littleton, Bellen, & Perin, 1993). Immunolabeling was performed as described in Aso et al. (2014). Additional details on reporter constructs are also described in this publication. A minimum of two female brains and three CNSs (a CNS is a brain plus ventral nerve cord, or VNC) per stable split line were immunolabeled and mounted in DPX. Detailed protocols are available at: https://www.janelia.org/project-team/flylight/protocols (see “IHC—Anti‐GFP,” “IHC—Polarity Sequential,” and “DPX mounting”). Imaging conditions for each brain and corresponding VNC are identical, so the intensity seen in the brain and VNC for each line accurately reflects the relative levels of expression in these two regions. Microscope settings were adjusted as necessary in different lines.

### 2.4 Antibody characterization

Primary antibodies used in this study are presented in Table 4. Several assays document the specificity of the anti‐Bruchpilot (a presynaptic active zone protein; BRP) antibody, nc82 (RRID: AB_2314866). Wagh et al. (2006) demonstrate (a) nc82 localization at presynaptic active zones, (b) specificity of anti‐nc82 in Western blot analysis, and (c) correspondence in nc82 localization and GFP label in tissues in which untagged and GFP‐tagged *bruchpilot* were ectopically expressed in the wing imaginal disc, trachea and epidermal cells. Furthermore, they show a correspondence between GFP and endogenous BRP patterns when a panneural driver is used to drive GFP‐tagged *bruchpilot*. Brp expression is lost in *brp* mutants and rescued by expression of BRP in these mutants (Kittel et al., 2006). Hamanaka and Meinertzhagen (Hamanaka & Meinertzhagen, 2010) report the distribution of nc82 at neuromuscular junctions and *Drosophila* photoreceptor synapses.

The antibodies used to detect the three epitope‐tagged smGFPs (Viswanathan et al., 2015) used for the MCFO studies (rat anti‐FLAG, rabbit antihemagglutinin, and mouse anti‐V5) are widely used commercial antibodies. Their specificity is indicated by the fact that the observed expression patterns differ between GAL4 lines, arguing against staining being directed against endogenous epitopes, but are reproducible for a given GAL4 line. Two secondary antibodies were used in the polarity protocol: ATTO647N goat antirat IgG (1:150; H&L; Rockland #612–156‐120) and CY3 goat antirabbit (1:1,000; Jackson Immuno Research #111–165‐144).

### 2.5 Immunohistochemistry, clearing, and mounting

A detailed protocol for MCFO immunohistochemistry and mounting is presented in Nern et al. (2015) and, for polarity, in Aso, Hattori, et al. (2014). There are subtle differences between the two protocols. The specifics are omitted here; the referenced articles should be consulted for successful tissue preparation. Following dissection in Schneider's medium, tissue was fixed in paraformaldehyde (PFA) diluted in Schneider's medium. Samples were then rinsed in PAT3 (Triton X‐100/bovine serum albumin/phosphate‐buffered saline), blocked in normal goat serum (NGS) and then, for MCFO brains, incubated in: mouse anti‐nc82 (Developmental Studies Hybridoma Bank, University of Iowa; RRID: AB_2314866) (Hofbauer et al., 2009; Wagh et al., 2006); rabbit anti‐HA (1:300; Cell Signaling Technology; RRID: AB_1549585); and rat anti‐Flag (1:200; Novus Biologicals; RRID:AB_1625981). Antibodies were diluted in NGS/PAT3. For MCFO, following rinses in NGS/PAT3, samples were incubated in secondary antibodies, including in Alexafluor‐488 donkey antimouse (1:400; Jackson Labs), Alexafluor‐594 donkey anti‐rabbit (1:500; Jackson Labs), and Alexafluor‐647 donkey anti‐rat (1:300; Jackson Labs) in 3% NGS/PAT3. Following additional rinses in PAT3 and a block in normal mouse serum/PAT3, tissue was incubated in DyLight‐549 mouse anti‐V5 (1:500; AbD Serotec; AB_915420). For the tissue prepared for polarity, primary antibodies rat anti‐Flag (1:200; Novus Biologicals, Littleton, CO), rabbit anti‐HA (1:300; Cell Signaling Technology, Danvers, MA), and anti‐nc82 (1:33) were used, and secondary antibodies AlexaFluor‐647 donkey anti‐rat (1:300; Jackson Labs) and CY3 donkey antirabbit (1:500; Jackson Labs). Brains were subsequently washed in PAT3, then in PBS, and postfixed in PFA. Finally, tissue was rinsed in PBS, followed by a PAT3 rinse, and mounted within 3–5 days.

Tissue was dehydrated through an ethanol series, placed on poly L‐lysine‐coated No. 1 coverslips and then further dehydrated. Following xylene treatment, tissue was mounted in DPX (Sigma‐Aldrich, St. Louis, MO). DPX was allowed to dry for 2 days before imaging.

### 2.6 Image acquisition and analysis

A Zeiss LSM 710 confocal microscope was used to collect images, using a Plan‐Apochromat 63×/NA1.4 oil immersion objective. Frame sizes were 1,024 × 1,024 pixels, voxel size 0.19 × 0.19 × 0.38 µm, zoom factor 0.8, and one frame average. While reference channel intensity was adjusted throughout the depth of the sample to maximize image quality, the gain and power were maintained at constant levels within each sample. The “Janelia Workstation” image‐viewing software (Murphy et al., unpublished data) was used to analyze confocal stacks.

### 2.7 Neuropil masks and brain alignment

Registration of data collected from individual brains into a standardized frame of reference can distort the morphology of neurons, so unaligned data was used to characterize the morphology of neurons described here. On the other hand, since aligning the data to a standard brain allows a comparison between brains, registered data was used for the color depth mask searches. Details for preparation of the standard brain used in this work (JFRC2013) can be found in Aso, Hattori, et al. (2014). Neuropil masks were generated using FluoRender (software that generates three‐dimensional images; https://bmcbioinformatics.biomedcentral.com/articles/10.1186/s12859-017-1694-9 or Wan et al., 2009, 2012), brains labeled with anti‐Brp, and a standard brain (JFRC2013). For details, refer to Aso, Hattori, et al. (2014).

### 2.8 Figure preparation

In cases in which the neuron intensity in MCFO images was below the threshold required for visibility in a publication, brightness, and contrast were increased in the Janelia Workstation. In several cases, anti‐nc82 label and neuron fragments from neurons that were not the focus of the figure, were “erased” using Adobe Photoshop (San Jose, CA). Brightness and contrast for each pair of brains and VNCs shown in Figure 2 were individually optimized; settings are identical for brains and corresponding VNCs within each sample.

## 3 RESULTS

### 3.1 Identification and characterization of split‐GAL4 lines

Most GAL4 lines drive expression in multiple cell types, sometimes making it difficult to isolate individual cells for anatomical analysis using the MCFO technique. Their activity in multiple cell types also makes these lines problematic for behavioral assays. To circumvent these limitations, the split‐GAL4 intersectional method (Luan et al., 2006) in combination with optimized vectors (Pfeiffer et al., 2010) was used to reduce the number of cell types targeted in a given line. A detailed description of this method and of the generation of the hemidriver collection of lines is provided in Materials and Methods.

Transgenic hemidriver lines (Dionne et al., 2018; Tirian & Dickson, 2017) carrying unique enhancer fragments that drive expression in cell types of interest were selected from two GAL4 collections (Jenett et al., 2012; Pfeiffer et al., 2008; Tirian & Dickson, 2017). Parental AD and DBD hemidriver lines judged to have overlapping expression patterns were crossed. A subset of these crosses is successful in producing lines in which the UAS reporter is expressed in a small number of cell types. The number of cell types that is observed in each intersection is cross‐specific and highly reproducible among the progeny of a given cross. The most useful combinations were made into stable split‐GAL4 lines that carry both the AD and DBD hemidriver constructs (see Methods). The “cleanest” split‐GAL4 lines for each cell type (i.e., those that show expression in the least number of cell types in the brain, optic lobes, and VNC) are presented in Table 2 and Figure 2. Images and confocal stacks for all lines listed in Table 2 can be accessed at http://www.janelia.org/split-GAL4.

The split‐GAL4 lines provided in Table 2 all drive expression in fewer cell types than the original GAL4 lines, but not all are specific for a single cell type. In some cases, this is due to the limited number of AD and DBD hemi‐drivers (the parent lines) in the available collections. In the case of the NO_3_M and NO_3_P cells, we were able to design many split‐GAL4 lines that are expressed in both of these cell types, but we could not identify enhancers that are uniquely expressed in each of these cell types; either they do not exist or the lines in the GAL4 collection do not target them.

Three analyses were used to characterize the morphology, polarity, and population size of the cells targeted by these lines. The first, the MCFO method, is a selective approach that stochastically labels small populations of cells, providing insight into cellular morphology and neuropils targeted by a given cell type; it does not, however, reveal the population‐wide projection pattern for that cell type.

Polarity data, generated in the second type of analysis in which cell type‐specific split‐GAL4 lines are used to drive expression of reporter constructs that target membranes and presynapses, supplements the MCFO data in that it provides molecular confirmation of the morphology‐derived polarity of neurons obtained using the MCFO methodology (not shown). These polarity data rely on the correct targeting of an exogenous fusion protein and can overpredict presynaptic sites in cases where expression levels are too high (Aso, Hattori, et al., 2014). Although the expression levels of this protein are titrated in this system, caution should still be exercised in interpreting these patterns. Similarly, while the MCFO anatomical data also has its limitations, it can reliably identify the predominant arbor type. Since neither of these methods provides the accuracy that can be achieved using electron microscopy (EM), and since arbors often have both inputs and outputs, EM‐level resolution will be required to establish the true percentage of spines and boutons for each terminal.

In the third method of analysis, brains were prepared using the Chrimson‐Venus marker to reveal the expression patterns (Figure 2 and http://www.janeliaorg/split-GAL4) of the GAL4 lines listed in Table 2. These samples reveal both the approximate population size of a given neuron type as well as each cell type's projection pattern. We observed minimal brain‐to‐brain variability in the number of cells in some lines. The variability is due to loss of cell bodies during dissection, stochasticity in gene expression, difficulty in resolving individual soma when they were tightly clustered, and perhaps some variability in neural composition between individuals. The cell counts provided are therefore not precise population sizes. Taken together, these three labeling techniques provide a comprehensive description of each cell type analyzed.

### 3.2 Nomenclature

The nomenclature system used here includes three features that unambiguously identify a neuron so that it can be recognized by its name: the structures targeted by the neuron; the primary neurite's path relative to the midline; and the polarity, deduced from the anatomy of the arbor. A detailed description of this nomenclature system can be found in Wolff et al. (2015); several points are reiterated here to assist in interpreting the neuron names presented in the results.

Central complex neurons connect up to three structures within the central complex and, in some cases, more if they also project outside the central complex. Each class of neurons follows a stereotyped projection pattern as it links these structures. Some neuron classes maintain ipsilateral paths through their targeted neuropils whereas others cross the midline. The crossover point—between the first and second or second and third neuropils—is a conserved feature of the neuron class. This midline crossover point and the locations of the arbors relative to one another are important anatomical features of the neuron and are therefore described within the neuron's name. The abbreviations “i” for ipsilateral, “c” for contralateral, and “ic” for instances when a projection arborizes in both the ipsi‐ and contralateral counterparts of a structure, are appended to all but the first neuropil in the neuron's name to indicate their location relative to the first neuropil. For example, in the hypothetical neuron “A‐Bi‐Ci‐Dc,” the arbors in neuropils B and C are on the same side of the midline as A's arbor, the primary neurite crosses the midline between neuropils C and D, and the arbor in structure D is in the contralateral hemisphere.

It is generally possible to distinguish between the two basic arbor morphologies of a neuron at confocal microscope‐level resolution, although EM studies will be required to quantitatively describe all arbors. These two morphologically distinct arbors, which resemble thin fibers and blebs, provide insight into the neuron's polarity and direction of information flow: the fine, thin arbors are typically associated with functional input whereas the bleb‐like boutons are typically recognized as the functional output of a neuron. These anatomical features are included in the nomenclature system used here. The abbreviations “s,” for terminals that appear to be predominantly thin spines, “b” for terminals that appear to consist predominantly of boutons, and “s.b” for obviously mixed terminals, are appended to each structure in the neuron's name. In the hypothetical neuron A.s‐Bi.s.b‐Ci.b‐Dc.b, neuropil A has primarily spines in its arbor, B is a mixture of both spines and boutons, and the arbors in neuropils C and D consist primarily of boutons.

One change to the nomenclature system used in Wolff et al. (2015) is being adopted here. To conform to the standard used in other species, neuropils with arbors that are predominantly spiny in morphology (presumed input regions) are listed first, obviously mixed terminals are next, followed last by presumed output regions.

Finally, the boundaries between some adjacent neuropils were defined based on subtle morphological features (Ito et al., 2014) that may not necessarily coincide with functional differences. The arbors of some neurons extend beyond these nebulous boundaries. For example, some neurons discussed below have extensive arbors in the LAL and a few fine terminals from these arbors extend minimally into the adjacent crepine (CRE). In an effort to both avoid confusion and simplify the names of neurons with such minimal excursions into adjacent neuropils, the name of the neuropil with minor representation is not included in the neuron's name. A comprehensive anatomical description that includes these details is, however, included in the text.

### 3.3 Recommended abbreviations for neuron names

The nomenclature used in this publication and in Wolff et al. (2015) is comprehensive but too cumbersome for reiterative use in papers and talks, so a set of standardized abbreviations is presented in Table 5. In an effort to keep the abbreviations as brief as possible, only critical information is retained. For example, FB layers and NO designations (1, 2, and 3) are excluded from the PB‐FB‐NO contractions since compartment information, such as anterior, dorsal, etc., uniquely identifies these cell types and is included. This abridged nomenclature is consistent with, and builds upon, a loose convention established by several labs working on central complex neurons, and it evolved from discussions among several individuals working on these neurons (see Acknowledgments). The following rules were used to derive the abbreviations in Table 5.

1. Central complex structures are abbreviated with a single capital letter that is the first letter of the neuropil's name (PB = P, FB = F, EB = E, NO = N, AB = A). Structures that are targeted by central complex neurons and that have unique first letters are similarly abbreviated (LAL = L, GA = G, CRE = C, ROB = R).
2. The remaining neuropils follow the three‐letter designations presented in Ito et al. (2014). To avoid confusion with the single letter notations of sequential neuropils in the abbreviation (see rule 5), only the first letter of the three‐letter code is capitalized (e.g., Slp, which, if SLP, could be confused for an unknown neuropil “S,” followed by the LAL, followed by the PB).
3. The field has established P‐EG and E‐PG as the abbreviations for the neurons PB_G1–9_.s‐EBt.b‐D/V GA.b and PB_G1–8_.b‐EBw.s‐D/V GA.b, respectively. These abbreviations will remain as such.
4. Neuropil subdomain information is included only when necessary to distinguish between otherwise identical abbreviations. In these cases, the first letter of the subdomain's name is appended as a subscripted capital letter. For example, the canal P‐EG, PB_G1–9_.s‐EBc.b‐D/V GA.b, is abbreviated P‐E_C_G, and the gall tip E‐PG cell, PB_G9_.b‐EB.P.s‐GA‐t.b, is abbreviated E‐PG_T._ (Note the original nomenclature system did not list input neuropils first, but the gall tip cell is, in fact, an E‐PG cell.) In these two cases, the subscripted “C” and “T” disambiguate these two cell types from the P‐EG and E‐PG noted in #3, above.
5. Apparent input (spiny morphology) neuropils are listed first followed by neuropils with mixed input and output followed last by apparent output neuropils (boutons).
6. Input, mixed, and output neuropils are separated with hyphens.
7. Variants that are not morphologically distinct are assigned arbitrary, nonsubscripted numerals that follow the designated abbreviation (e.g., P‐EN1 and P‐EN2).
8. Cells with formal names that convert to pronounceable acronyms will adopt those acronyms (e.g., Delta7).

**Table 5:** Neuron abbreviations

	Abbreviation
**PB neurons**	
PB_G2‐9_.s‐FBℓ1.b‐NO_3_P.b	P‐FN_P_
PB_G2‐9_.s‐FBℓ1.b‐NO_3_M.b	P‐FN_M_
PB_G2‐9_.s‐FBℓ2.b‐NO_3_A.b	P‐FN_A_
PB_G2‐9_.s‐FBℓ3.b‐NO_2_D.b	P‐FN_D_
PB_G2‐9_.s‐FBℓ3.b‐NO_2_V.b	P‐FN_V_
PB_G2‐9_.s‐EBt.b‐NO_1_.b	P‐EN1, P‐EN2
PB_G1‐9_.s‐EBt.b‐D/V GA.b	P‐EG
PB_G1‐8_.b‐EBw.s‐D/V GA.b	E‐PG
PB_G1‐9_.s‐EBc.b‐D/V GA.b	P‐E_C_G
PB_G9_.b‐EB.P.s‐GA‐t.b	E‐PG_T_
PB.s‐FBℓ6.b.ℓ3.s‐V GA‐s.b	P‐F‐G_S_
PB_G1‐8_.s‐FBℓ3,4,5.s.b‐ROB.b	P‐F‐R
PB_G1‐7_.s‐FBℓ2.s‐LAL.b‐CRE.b	PF‐LC
PB.b‐LAL.s‐PS.s	LPs‐P
PB_G6‐8_.s_G9_.b	P_6‐8_‐P_9_
PB18.s‐GxΔ7Gy.b	Delta7
B_G1/2‐9_.b‐SPSi.s	Sps‐P
PB_G2‐9_.b‐IB.s.SPS.s	IbSps‐P
**NO neurons**	
LAL.s‐GAi.s‐NO_1_i.b	LG‐N
LAL.s‐NO_2_i.b	L‐N
LAL.s‐NO_3_Ai.b	L‐N_A_
LAL.s‐CREi.s‐NO_3_P/Mi.b	LC‐N_PM_
LAL.s‐CREc.s‐NO_3_Pc.b	LC‐N_P_
**AB neurons**	
SLP.s‐AB.b	Slp‐A
AB.s.b‐FBℓ8.b	A‐F
SLP.s‐AB.b‐FBℓ8.b	Slp‐AF

### 3.4 NO neurons

The classification of a neuron as a PB, FB, EB, or NO cell is somewhat arbitrary since central complex neurons arborize in one to three structures of the central complex and could therefore be classified by any one of the three targeted neuropils. We began our studies of the central complex with the PB (Wolff et al., 2015), so all neurons that were found to arborize in the PB were assigned to the PB class, even though many of them project to additional structures within and outside of the central complex. Neurons presented here as “NO neurons” exclude both those described previously as PB neurons that also arborize in the NO, for example, the PB‐FB‐NO and PB‐EB‐NO neurons (Lin et al., 2013; Wolff et al., 2015), as well as tangential FB neurons that arborize extensively throughout one layer of the FB and also send more restricted projections to the NO (for example, see Hanesch, Fischbach, & Heisenberg, 1989). In the discussions that follow, “NO neuron” refers exclusively to those neurons described in the results.

**Figure 3:**
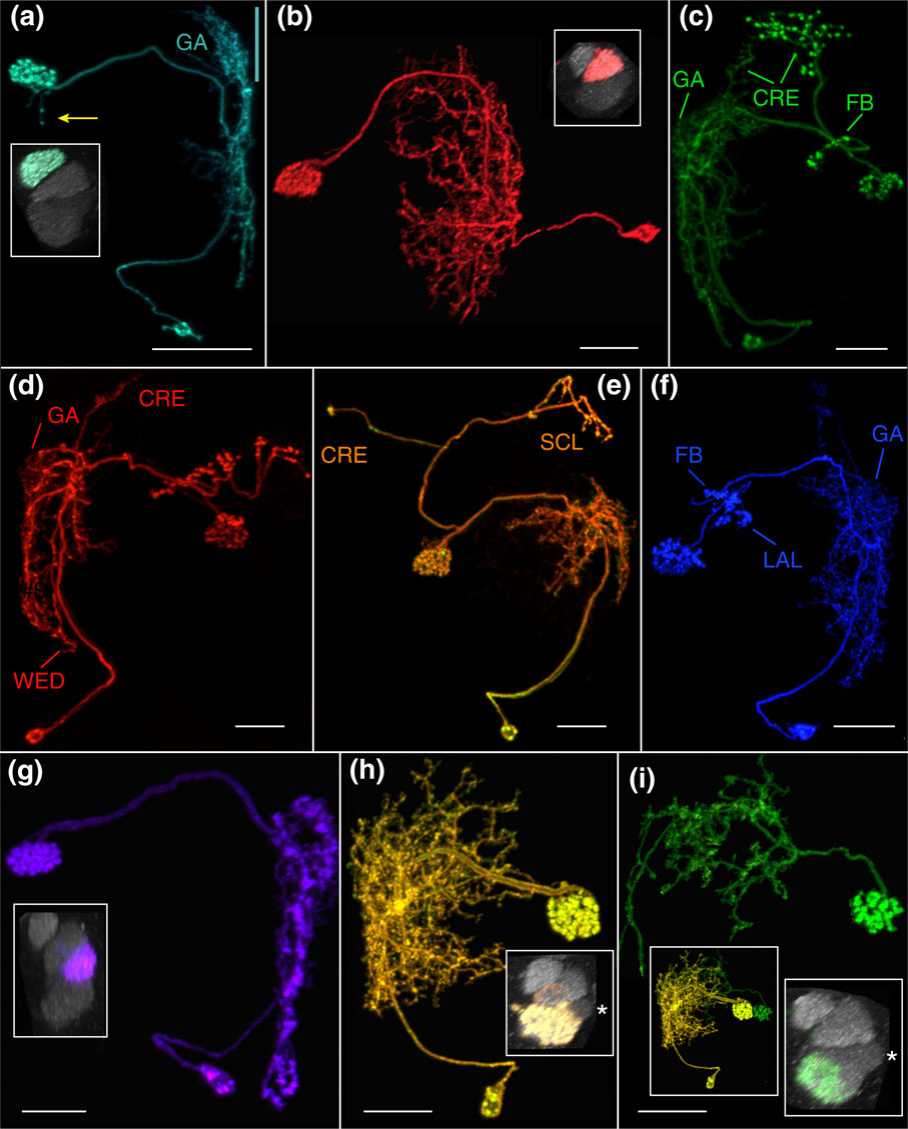
Nodulus neurons. (a) LAL.s‐GAi.s‐NO_1_i.b, the only NO_1_ neuron identified to date. The arbors in the GA and LAL are predominantly input, populate the entire gall (length is highlighted by green line) and just the lateral margin of the LAL. Inset, sagittal view of noduli. NO_1_, the dorsal nodulus, receives the output from this neuron. GA = gall. Scale bar = 30 μ. [MCFO image from line R76E11]. (b) LAL.s‐NO_2_i.b neuron. The LAL arbor is more robust than the NO_1_ cell shown in (a). Inset, both dorsal and ventral compartments of NO_2_, the medial nodulus, are filled with boutons. Scale bar = 20 μ [MCFO image from line R41H08]. (c) LAL.s‐GAi.s‐CREi.s‐NO_2_i.b‐FBℓ3i.b‐CREi.b neuron. The presumed output arbor in the crepine is substantial whereas just a few fine, thin terminals project into a different region of this neuropil and represent apparent offshoots from the LAL arbor, which tracks primarily along the lateral margin. The GA arbor fills both domains of the GA. CRE = crepine. Scale bar = 20 μ [MCFO image from line SS04682]. (d) LAL.s‐GAi.s‐CREi.s‐NO_2_i.b‐FBℓ3.b neuron. The FB arbor most likely should extend the full span of FBℓ3. The CRE arbor in this cell is similar to the input CRE arbor of the cell in (c) in that it is minimal and appears to extend from the more elaborate LAL arbor. CRE = crepine; WED = wedge. Scale bar = 20 μ [MCFO image from line SS04682]. (e) LAL.s‐NO_2_i.b‐SCLi.b‐CREc.b neuron. Minimal fine terminals extend into the GA and only one bouton populates the contralateral CRE. SCL = superior clamp. Scale bar = 20 μ. [MCFO image from line SS04682]. (f) LAL.s‐GAi.s‐NO_2_i.b‐FBℓ3i.b‐LALi.b neuron. Either the FB arbor is columnar or it is also artificially truncated, as is expected to be the case with the neuron shown in panel d. Scale bar = 20 μ [MCFO image from line SS04682]. (g) LAL.s‐NO_3_Ai.b neuron. This LAL arbor's flocculent texture distinguishes it from the other NO neurons. Inset, as indicated by the cell's name, only the anterior compartment of NO_3_ (NO_3_A) is targeted by this cell. Scale bar = 20 μ [MCFO image from line R12G04]. (h) LAL.s‐CREi.s‐NO_3_P/Mi.b neuron. The distribution of the spiny arbor is widespread throughout the LAL and reaches into the CRE, which lies anterior to the bulk of the LAL arbor and is therefore not identified in the figure. As with all of the NO cell types described here, the nodulus arbor is predominantly composed of boutons, and, in this case, fills both the medial and posterior NO_3_ compartments, as shown in the inset. The asterisk identifies NO_3_A in the inset. Scale bar = 20 μ [MCFO image from line SS04689]. (i) This LAL.s‐CREc.s‐NO_3_Pc.b neuron is unique among the identified NO neurons in that it crosses the midline between the LAL and NO. It is from the same brain as the neuron shown in (h); both neurons are shown in left inset. The distinction between these two neurons, particularly the ipsilateral versus contralateral circuitry, is evident in the left inset. Right inset illustrates this neuron's absence from NO_3_M [note the gap between the filled NO_3_P neuropil and NO_3_A, marked by an asterisk, a gap that is filled with the yellow arbor in the inset in (h)]. Scale bar = 20 μ [MCFO image from line SS04689]

**Figure 4:**
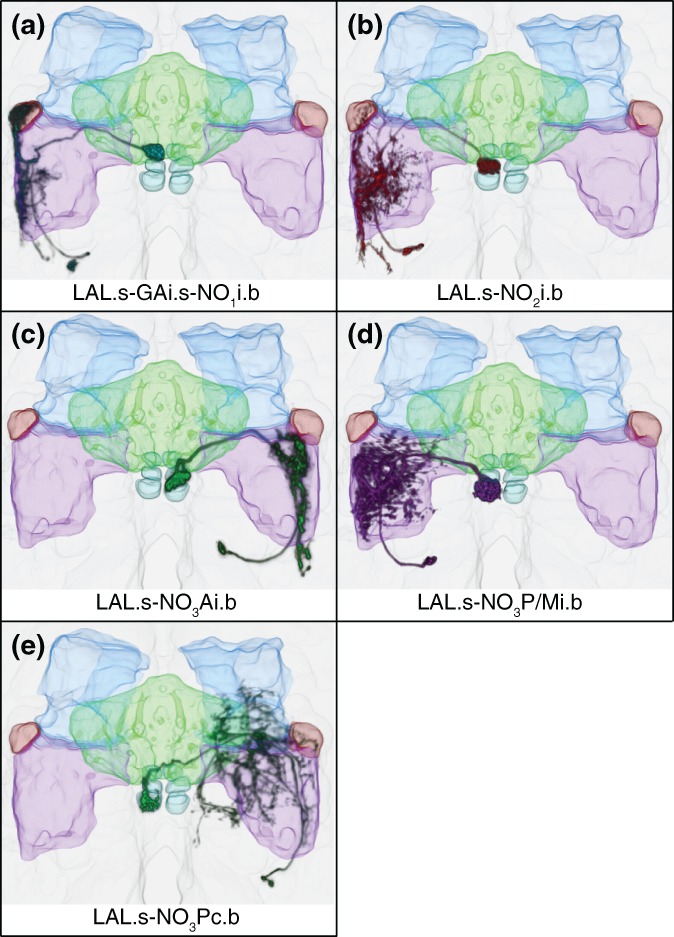
Registered images of NO cell types. Examples of the five most commonly seen NO neurons were aligned to standard brain JRC2013 and rendered in three dimension using FluoRender (https://bmcbioinformatics.biomedcentral.com/articles/10.1186/s12859-017-1694-9; (Wan et al., 2009, 2012). FB = green; NO = cyan; GA = maroon; LAL = lilac; CRE = blue. Scale bar = 20 μ. (a) LAL.s‐GAi.s‐NO_1_i.b. (b) LAL.s‐NO_2_i.b. (c) LAL.s‐NO_3_Ai.b. (d) LAL.s‐CREi.s‐NO_3_P/Mi.b. As noted above (Figure 3H), the minimal fine terminals that arborize in the crepine are not visible from this angle. (e) LAL.s‐CREc.s‐NO_3_Pc.b

This work identified as many as nine distinct “nodulus neurons” (Figure 3), with the most frequently seen types illustrated in Figure 4. The common themes of the NO neurons described here are: (a) they project to only one nodulus, the “left” or “right,” of a pair; (b) the arbors in the noduli appear to be predominantly presynaptic: only boutons are evident in light‐level, confocal images; (c) presumed input, based on anatomy, comes almost entirely from the LAL; (d) four of the five most commonly seen classes of NO neurons are exclusively ipsilateral, targeting structures only in either the left or right hemisphere. The morphology of the LAL arbors and the subregions they occupy within the LAL are largely distinct for each of the NO neuron types.

#### 3.4.1 NO_1_‐targeted neuron

Just one cell type that targets the dorsal nodulus (NO_1_) has been identified, the ipsilateral LAL.s‐GAi.s‐NO_1_i.b neuron (*n* > 81; Figures 3a and 4a). As indicated by this neuron's nomenclature, light‐level analysis indicates that this neuron has primarily spiny arbors in the LAL and GA and predominantly boutons in NO_1_, suggesting information is relayed from the LAL and GA to the NO. In some cases, one to two boutons extend from NO_1_ into NO_2_ or NO_3_ (Figure 3a, arrow). The bulk of the LAL arbor is confined to the lateral margin of the LAL (Figure 4a) and its length is somewhat variable. The gall arbor fills both its dorsal and ventral compartments.

#### 3.4.2 NO_2_‐targeted neurons

The NO_2_‐targeted neurons identified to date project to both the dorsal and ventral subcompartments of the medial nodulus, NO_2_. This distribution is implied in the names of each of these neurons by the absence of the “D” and “V” (for dorsal and ventral) designations. Five NO_2_ cell types are described, although four are rare. Although the sample size for these four neurons is small, their morphologies differ significantly from the most common cell type, LAL.s‐NO_2_i.b (below), and they display features that distinguish them from one another. These morphological criteria suggest that these four neurons are strong candidates to be bona fide distinct neuronal types; they are therefore described here.

##### LAL.s‐NO_2_i.b

The ipsilateral LAL.s‐NO_2_i.b neuron has predominantly spiny branches in the LAL whereas the NO_2_ arbor is a densely packed bundle of boutons (*n* > 52; Figures 3b and 4b). The LAL arbor is more proliferative than the NO_1_ neuron's LAL arbor. Its dorsal boundary often creeps slightly into the adjacent CRE (Figure 4b). The arbor's intrusion into the CRE is minimal and therefore not included in the neuron's name.

##### Four putative cell types

Four additional “putative” NO_2_ cell types are described below. Each of these possible cell types has been seen just one or two times unless otherwise noted, all in the same split‐GAL4 line, SS04682, so we cannot be certain that they represent bona fide cell types rather than cells with aberrant projections. If this is the case, it suggests the commonly seen LAL.s‐NO_2_i.b cell is highly prone to errors in line SS04682 and these putative cells may represent developmental mistakes. However, this possibility seems less likely since the morphological features of these cells are robust and elaborate—not typical of the projections we have occasionally seen in other lines that resemble developmental miswiring. Alternatively, the scarce number of sightings may simply reflect that these neuron types are rare or infrequently targeted with the stochastic MCFO labeling approach. (MCFO was performed on >100 brains of this line.)

Of the putative cell types described below, the first is most dissimilar to the rest and the remaining three share several features in common (discussed below). Definitive classification of these putative cell types awaits dense EM‐level reconstruction of this neuropil.

##### LAL.s‐GAi.s‐CREi.s‐NO_2_i.b‐FBℓ3i.b‐CREi.b

The LAL.s‐GAi.s‐CREi.s‐NO_2_i.b‐FBℓ3i.b‐CREi.b (Figure 3c) has apparent input from the LAL, GA (which it fills) and, minimally, the CRE. Apparent output is in the CRE, FB, and NO_2_. In the example shown, the FB arbor occupies just a portion of FBℓ3. The partial FBℓ3 arbor is likely a driver‐induced flaw since an unusual but common phenomenon seen in brains from the line in which this cell was documented, SS04682, is that many FB arbors from cell types not discussed here extend throughout only a variable portion of the layer whereas these same cell types are not “truncated” in the FB in other lines.

##### LAL.s‐GAi.s‐CREi.s‐NO_2_i.b‐FBℓ3.b

With the exception of the FB arbor in this cell, this is an ipsilateral neuron (*n = * 9; Figure 3d). The LAL and gall arbors are predominantly spiny, and the gall arbor projects throughout the gall. One small shoot extends ventrally into the wedge (WED) and a second, distinctive shoot reaches dorsally into the CRE. Boutons fill the medial nodulus (NO_2_) and just the ventral portion of NO_1_ as well as about two‐thirds of the width of layer 3 of the FB. As with the previously described cell type, it is possible that this arbor should extend the span of FBℓ3 but is truncated due to driver toxicity. The most obvious morphological difference between this cell and the LAL.s‐GAi.s‐CREi.s‐NO_2_i.b‐FBℓ3i.b‐CREi.b cell described above is the extensive array of boutons that is absent in the CRE of this cell (LAL.s‐GAi.s‐CREi.s‐NO_2_i.b‐FBℓ3.b).

##### LAL.s‐NO_2_i.b‐SCLi.b‐CREc.b

This cell type is obviously distinct from the other four putative NO_2_ neurons. The most distinguishing features are the long projection to the contralateral crepine (CRE.c) that terminates in a single bouton and the bifurcating trajectory to the ipsilateral superior clamp (SCL), where it terminates in a small number of boutons (Figure 3e). The LAL arbor of this cell is much less robust than that of LAL.s‐NO_2_i.b (Figure 3b). The LAL arbor is primarily spiny and a few branches reach into the gall.

##### LAL.s‐GAi.s‐NO_2_i.b‐FBℓ3i.b‐LALi.b

This putative cell type (Figure 3f) shares several features in common with the other three putative cell types: bouton‐rich arbors in both subcompartments of NO_2_, a spiny arbor in a large portion of the LAL and the entire GA, and boutons in a fraction of FBℓ3. This cell type also has a separate, small arbor of primarily boutons in the LAL, close to the FB arbor, and its NO_2_ arbor appears to send small shoots into NO_3_A and the vest (a large bilateral neuropil ventral to the FB), although more samples are needed to confirm this observation. This cell most closely resembles LAL.s‐GAi.s‐CREi.s‐NO_2_i.b‐FBℓ3.b (Figure 3d), and while it is plausible that they are variants of the same cell, this seems less likely given the absence of the dorsal‐projecting shoot that targets the CRE as well as the greater number of fine branches in the LAL arbor in the LAL.s‐GAi.s‐NO_2_i.b‐FBℓ3i.b‐LALi.b cell.

#### 3.4.3 NO_3_‐targeted neurons

Three subcompartments comprise the large, ventral nodulus, NO_3_: NO_3_A, NO_3_M, and NO_3_P (Figure 1d) (Wolff et al., 2015). Three neurons that arborize in NO_3_ have been identified. Based on morphology, likely input regions for these three neurons include the LAL and, in two cases, a small amount of input from the CRE. The nodulus arbors for these cells are rich in boutons, suggesting this neuropil is primarily receiving input from the NO_3_ neurons.

##### NO_3_A

The LAL.s‐NO_3_Ai.b neuron is the only currently identified cell type that targets NO_3_A (*n* > 86; Figures 3g and 4c). As with LAL.s‐GAi.s‐NO_1_i.b (Figure 3a) and LAL.s‐NO_2_i.b (Figure 3b), this is also an ipsilateral neuron. Similar to the LAL.s‐GAi.s‐NO_1_i.b cell type, the LAL arbor is confined to the lateral margin of this neuropil, running the length of the LAL (Figure 4c). An occasional bouton projects from the NO_3_ arbor into the NO_2_ subcompartment (not shown).

##### NO_3_P/M

The ipsilateral LAL.s‐CREi.s‐NO_3_P/Mi.b (*n* > 107; Figures 3h and 4d) resembles LAL.s‐NO_2_i.b (Figure 3b) in its gross morphology, primarily in its expansive dendrite‐rich LAL network. The NO_3_P/M neuron also has dendrites in the CRE and occasional spines reach into the vest. This neuron has a dense population of boutons in both the posterior and medial compartments of NO_3_ (inset).

##### NO_3_P

The LAL.s‐CREc.s‐NO_3_Pc.b neuron (*n* > 73; Figures 3i and 4e) exhibits one notable feature that distinguishes it from the other four classes of common NO‐LAL neurons described here: it is not an ipsilateral neuron. Instead, with predominantly spiny arbors in the LAL and boutons in the contralateral posterior domain of NO_3_ (NO_3_P), the morphology suggests the predominant input comes from the LAL (with a smaller contribution from the CRE) on one side of the brain and is delivered to the contralateral nodulus. Unlike the LAL.s‐CREi.s‐NO_3_P/Mi.b neuron described above, only the NO_3_P subcompartment is targeted by this cell (Figure 3i, right inset). The LAL arbor in this cell type is more sparse than that in the LAL.s‐NO_2_i.b and LAL.s‐CREi.s‐NO_3_P/Mi.b cell types, but it covers roughly the same region (Figure 3i, left inset).

### 3.5 AB anatomy and neurons

#### 3.5.1 The asymmetrical body is a distinct, bilateral neuropil that is asymmetric in size and bilaterally innervated

**Figure 5:**
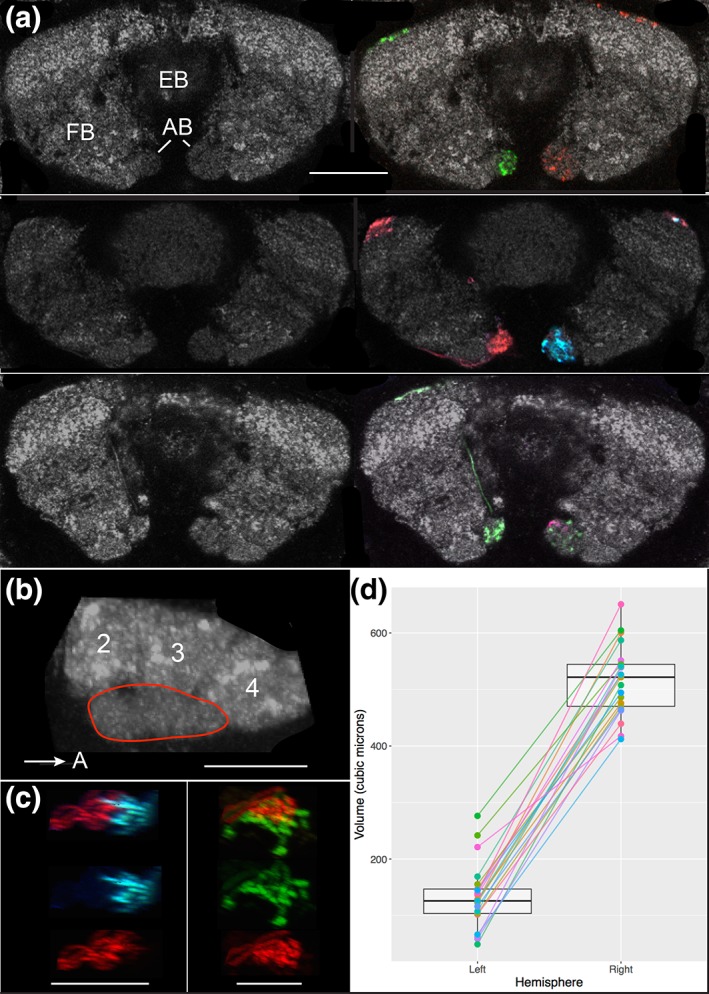
The asymmetrical body is a bilateral and asymmetric structure. (a) Confocal images illustrate the asymmetrical body is a bilateral structure and that the fly's right AB (on right in figure) is larger than its left AB. Frontal sections through the FB and EB from three brains are shown. The reference channel (anti‐Brp) is shown in the left panel of each set and the corresponding signal (neuron) channels are shown in the right panels. The signal channels identify the AB neuropils and demonstrate that both the left and right ABs are innervated. FB = fan‐shaped body; EB = ellipsoid body; AB = asymmetrical bodies. Scale bar = 20 μ [MCFO images from lines SS00241 and R41H08]. (b) The AB, outlined in red, is oblong in shape and is nestled ventral to teeth 2, 3, and 4 of the FB (see Wolff et al., 2015 for description of FB teeth). This sagittal view illustrates the length of the right AB. Scale bar = 10 μ. (c) The AB may be a compartmentalized structure. Two instances are shown in which the asymmetrical body is targeted by two neurons with nonoverlapping arbors (sagittal view). Neuron types are described in detail below. The arbors are shown together (top) and separately (middle and lower panels). In the left series of photos, AB.s.b‐FBℓ1c.b‐FBℓ8c.b (blue) and AB.s.b‐FBℓ8i.b (red) neurons occupy adjacent regions of the AB. Scale bar = 20 μ. In the right panel, AB.s.b‐FBℓ1i.b‐FBℓ8i.b and AB.s.b‐FBℓ1c.b‐FBℓ8c.b target nonoverlapping dorsal and ventral regions of the AB. Scale bar = 20 μ [MCFO images from line VT020016]. (d) Paired dot plot of left and right AB volumes (measured in μ^3^) overlaid on a box plot. While there is a great deal of variability in AB volume both between brains and between hemispheres, the left AB is consistently on average 25% of the volume of the right AB, but ranged from 10 to 53%

The analysis presented here indicates that the AB, while asymmetric in morphology, is present in both hemispheres in all brains (*n* > 167; Figure 5). Furthermore, both the left and right ABs are innervated (Figure 5a, right member of each pair). The AB is evident in confocal micrographs of both male and female wild‐type brains immunolabeled with anti‐Brp, including assorted GAL4 lines (*n = * 135 female plus 20 male) as well as the Berlin (*n = * 5 female), DL (Dickinson Lab; *n = * 3 female), and Oregon R (*n = * 4 female) wild‐type strains.

The AB is positioned adjacent to layer 1 of the FB and juxtaposes the medial–ventral boundary of the FB (Figure 5a,b). These structures have round profiles mediolaterally and dorsoventrally, but are oblong in the anteroposterior axis (Figure 5b). The restriction of neuronal arbors to either the FB or AB suggests the AB is a distinct neuropil rather than an extension of the FB. This respect for the AB/FB boundary is typical. For example, Fas II staining is restricted to the AB (Pascual et al., 2004), as are arbors of the AB neurons (below). Likewise, FB (unpublished) and PB‐FB (Wolff et al., 2015) neuron arbors are confined to the FB. Despite the separation of arbors innervating the AB and FB, these neuropils are situated in close proximity to one another: The presynaptic active zones of the two neuropils, revealed by anti‐Brp immunolabeling, are only distinctly separate at confocal microscope resolution in the posterior region of the AB, not in the anterior region.

Several instances indicate a possible compartmentalization of this neuropil. In the two examples shown, two neurons stochastically labeled using MCFO target different regions of the AB (Figure 5c).

Although this work shows the AB is a bilateral structure, the two neuropils are asymmetric in size, with the right AB having a larger volume than the left. The right AB measures 10 μ in diameter at its widest point (posterior) versus 7 μ for the left, and 590 μ^3^ versus 205 μ^3^ in volume for the JRC2013 standard brain. The variance across brains and hemispheres is illustrated in the paired dot plot (Figure 5d). Of the 21 brains measured, the right AB volume ranged from 412 μ^3^ to 651 μ^3^ and the left from 49 μ^3^ to 276 μ^3^. On average, the right AB is four times larger in volume than the left AB. The median volumes are 126 μ^3^ (left AB) and 522 μ^3^ (right AB [*n = * 21], as illustrated in the box plot (Figure 5d). Given the asymmetry in size, the term “asymmetrical body” still appropriately describes this neuropil.

#### 3.5.2 AB neurons and neuron families

Three AB neurons or neuron families have been identified and are described below. These classes of neurons are primarily, but not exclusively, a conduit for information flow between the superior lateral protocerebrum (SLP), FB, and AB. No intrinsic AB neurons have been identified. The AB cell types described in this report innervate both the left and right AB. One or both ABs can be targeted by a single neuron, depending on the cell type. One cell type only innervates the left AB if the right AB is also innervated by the same neuron (see below). The AB neurons appear, based on anatomy, to primarily provide input to the AB, with two of the three AB neurons or families of neurons exhibiting predominantly boutons in the AB. Since the small, left AB is innervated, it is reasonable to conclude it is a functional neuropil.

##### The SLP‐AB neuron

**Figure 6:**
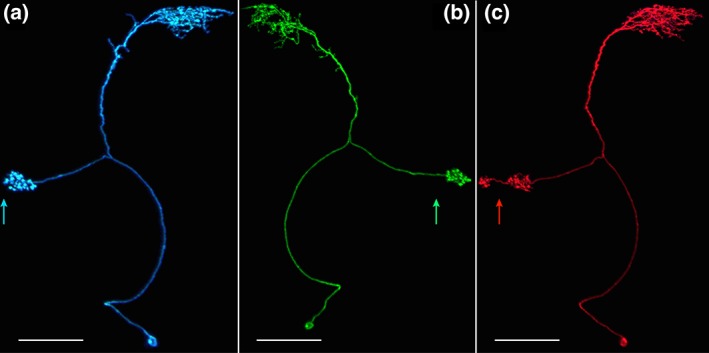
SLP‐AB neuron. This neuron exhibits a bias toward the right AB and comes in three forms. Arrows indicate the midline in each panel. (a) SLP.s‐ABi.b is the ipsilateral version with predominantly fine terminals in the SLP and boutons in the AB. Scale bar = 30 μ [MCFO image from line VT016127]. (b) SLP.s‐ABc.b is the contralateral form that only targets the right AB and therefore always the left SLP. The polarity is the same as described in a, above. Scale bar = 30 μ [MCFO image from line VT016127]. (c) SLP.s‐ABic.b, the dually innervated form. Apparent input from either the left or right SLP is delivered to both the left and right AB. Scale bar = 30 μ [MCFO image from line SS04458]

**Figure 7:**
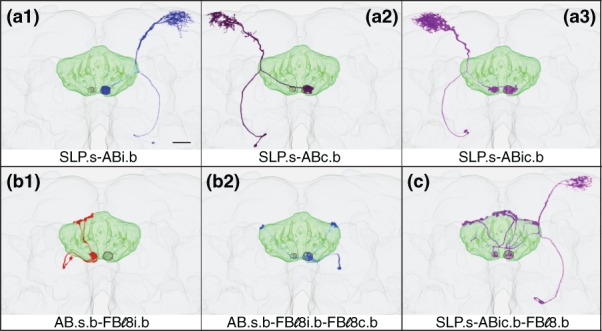
Registered images of AB neurons. Selected representative AB neurons were aligned to a standard brain (JRC2013) and rendered in three dimensions using the FluoRender software (https://bmcbioinformatics.biomedcentral.com/articles/10.1186/s12859-017-1694-9). Scale bar = 20 μ. (a1) SLP.s‐ABi.b. (a2) SLP.s‐ABc.b. (a3) SLP.s‐ABic.b. (b1) AB.s.b‐FBℓ8i.b. (b2) AB.s.b‐FBℓ8i.b‐FBℓ8c.b. (c) SLP.s‐ABic.b‐FBℓ8.b

The SLP.s‐AB.b cell type arborizes in just two neuropils, the SLP and AB. The arbors are predominantly spiny in the SLP and consist overwhelmingly of boutons in the AB. This neuron type exhibits three trajectories: an ipsilateral path (SLP.s‐ABi.b; Figures 6a and 7a1), a path that crosses the midline (SLP.s‐ABc.b; Figures 6b and 7a2), and one that arborizes in both the left and right ABs and the SLP in either the left or right hemisphere (SLP.s‐ABic.b; Figures 6c and 7a3). Interestingly, neurons exhibiting the first two pathways arborize exclusively in the right AB, so all SLP.s‐ABi.b information is relayed within the right hemisphere whereas SLP.s‐ABc.b‐transduced information crosses the midline from the left SLP to the right AB. The left AB receives information from the SLP only when the right AB also receives the same information, via the SLP.s‐ABic.b pathway.

The SLP.s‐ABic.b trajectory is less common than either the SLP.s‐ABi.b or SLP.s‐ABc.b trajectories; the latter two occur in approximately equal numbers. Furthermore, there is no strong bias toward the left or right SLP for the SLP.s‐ABic.b trajectory. One hundred fourteen MCFO‐labeled SLP‐AB neurons in line SS04682 were characterized as follows: right SLP, right AB (SLP.s‐ABi.b), *n = * 46; left SLP, right AB (SLP.s‐ABc.b), *n = * 40; left SLP, both ABs (SLP.s‐ABic.b), *n = * 18; and right SLP, both ABs (SLP.s‐ABic.b), *n = * 10.

These data indicate that the SLP‐AB neuron exhibits a strong asymmetric bias toward the right AB, but a similar strong right/left preference does not extend to the SLP. Furthermore, a single AB (always the right) is targeted more frequently than both simultaneously (75% vs. 25%, respectively, for SS04682). An additional 23 neurons labeled using MCFO, distributed among six stable split lines (SS04691, SS04692, SS04683, SS02822, SS004429, and SS04458), fell into the following classes: 7 SLP.s‐ABc.b (left SLP, right AB), 10 SLP.s‐ABi.b (right SLP, right AB), and 6 SLP.s‐ABic.b. Imaging data for these lines in which the entire population is labeled support the right AB bias for this neuron (not shown).

##### The AB‐FBℓ8 neuron family

**Figure 8:**
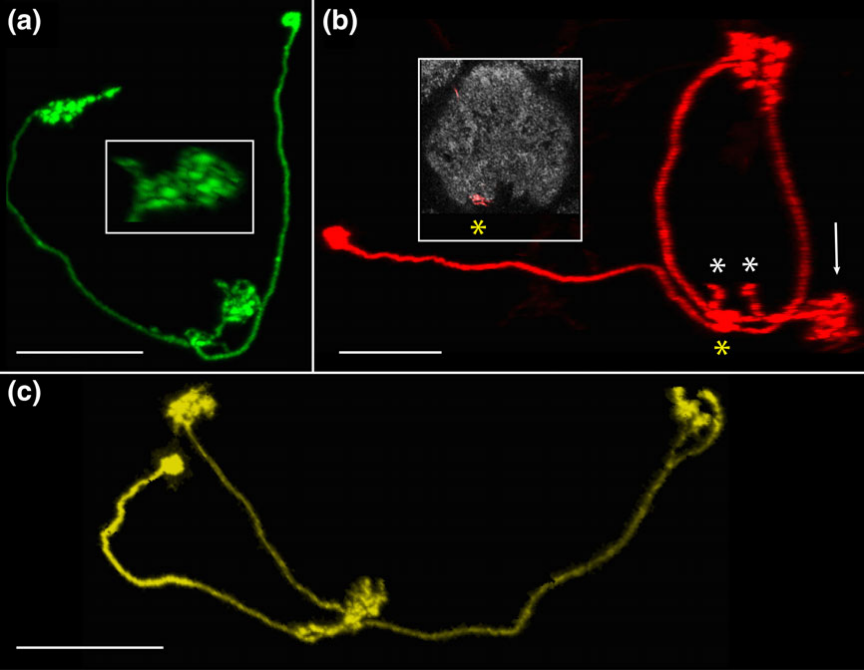
AB‐FBℓ8 neuron family. Three recurring forms of this family have been seen, and it seems likely additional subtly distinct forms exist. The common themes among members of this family are the mixed terminals in the AB, boutons in FBℓ8, and columnar arbors in the FB. (a) The “basic form” that defines this family: AB.s.b‐FBℓ8i.b. The cell shown here arborizes in the left AB. Inset: AB; note the presence of spines and boutons. Scale bar = 20 μ MCFO image from line SS02718. (b) The basic form shown in panel a plus a sparsely populated FBℓ1 and two, adjacent arbors in layer 8 of the FB: AB.S.B‐FBℓ1i.b‐FBℓ8i.b‐FBℓ8c.b. This neuron was rotated to enable the various arbors to be distinguished from one another. The FBℓ1 arbors are minimal, often just a single bouton or spine and rarely as many as five boutons. The AB and FBℓ1 arbors are virtually always in the same hemisphere. All asterisks identify boutons in FBℓ1; the yellow asterisk corresponds to the arbor in FBℓ1 in the inset. Arrow identifies the left AB. Scale bar = 20 μ [MCFO image from line SS02738]. (c) This family member, AB.s.b‐FBℓ8i.b‐FBℓ8c.b, projects two separate FBℓ8 arbors, one to each hemisphere, and one mixed arbor to the left AB. Scale bar = 20 μ [MCFO image from line SS00241]

The AB.s.b‐FBℓ8.b neuron comprises three sometimes subtly morphologically distinct forms (Figure 8). These differences can be so subtle, for example, one bouton in FBℓ1, that they may simply reflect the range of variation or stochastic nature of this cell type. In an effort to encompass the diversity in forms of this neuron, we designate it a “family.” All members of the family share several features in common: they all arborize in the AB and FBℓ8, the FB arbors are columnar, and the AB terminals are mixed, consisting of both boutons and spines. The presence of spines in the AB of this family is unique among the three AB cell types characterized to date and identifies them as the only output pathway (based on morphology) so far identified from this neuropil. Family members differ in the number of arbors in FBℓ8 and in whether or not they arborize in FBℓ1.

Three forms have been identified. (a) AB.s.b‐FBℓ8i.b, which has mixed terminals in the AB and apparent output in the ipsilateral layer 8 of the FB (*n = * 77, 33 of which arborize in the left AB, 43 in the right AB, and one in both ABs; Figures 8a and 7b1). (b) AB.s.b‐FBℓ1i.b‐FBℓ8i.b‐FBℓ8c.b (shown, Figure 8b), although sometimes lacks the contralateral FBℓ8 arbor (AB.s.b‐FBℓ1i.b‐FBℓ8i.b; not shown). This form is similar to that shown in Figure 8a but in addition, it has sparse output in the ipsilateral FBℓ1 and sends two projections to FBℓ8 (*n = * 11; 7 arborize in the left AB, 3 in the right AB, and 1 with output in the contralateral FB layers 1 and 8). (c) AB.s.b‐FBℓ8i.b‐FBℓ8c.b, which has mixed terminals in the AB and two apparent output arbors in FBℓ8, one in each “shoulder,” or lateral margin (*n = * 14; 9 target the left AB and 5 the right AB; Figures 8c and 7b2). The differences between these forms are subtle and may simply reflect the stochasticity of this cell type. (Cell counts are from lines SS00241, SS02218, SS02718, SS02738, SS02742, SS04417, and VT020016.)

##### SLP‐AB‐FBℓ8 neuron family

**Figure 9:**
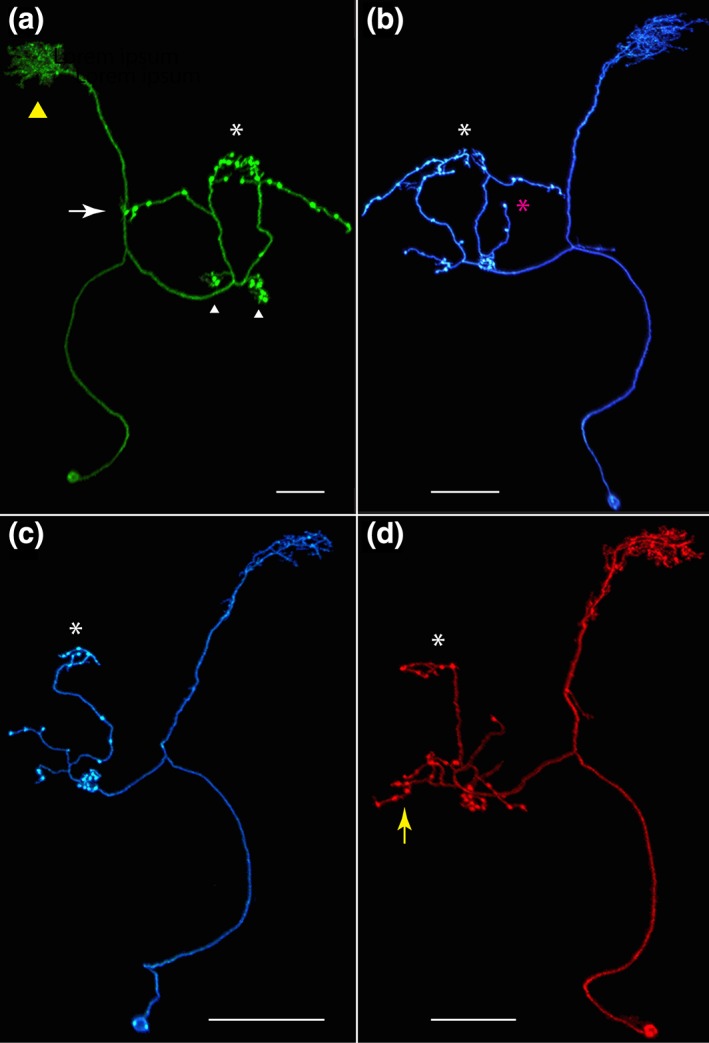
SLP‐AB‐FBℓ8 family. The range of overlapping phenotypes seen in this set of neurons links them together as a family. All neurons in the family exhibit spiny arbors in the SLP, boutons in one or both ABs, and boutons in FBℓ8. Additional neurites decorated with boutons project into various layers of the FB, and examples shown are named to reflect these features, although there are likely so many subtly different forms that the generic “SLP‐AB‐FBℓ8” neuron is more practical. White asterisks indicate midline. (a) The minimal features of this family of neurons are displayed by this SLP.s‐ABic.b‐FBℓ8.b neuron. Yellow arrowhead = SLP; white arrowheads = ABs; white arrow = FBℓ8. Scale bar = 20 μ [MCFO image from line SS02922]. (b) Additional boutons populate several FB layers, including layers 1, 4, 5, and 8 (e.g., red asterisk) in this family member. The characteristic thin fibers in the SLP are evident. Scale bar = 30 μ [MCFO image from VT060202]. (c) This SLPi.s‐AB.b‐FBℓ1.b‐FBℓ8.b neuron illustrates the more restricted FBℓ8 arbor described in the text. Scale bar = 40 μ [MCFO image from line VT060202]. (d) SLP.s‐ABi.b‐FBℓ1.b‐FBℓ2.b‐FBℓ5.b‐FBℓ8.b also has an FBℓ8 arbor that is constrained to the midline (white asterisk, which also identifies midline) and a more extensive FBℓ1 (yellow arrow). Scale bar = 30 μ [MCFO image from line SS04423]

The SLP.s‐AB.b‐FBℓ8.b neuron family has the least stereotyped morphology of all PB, NO, and AB neuron classes studied to date. The term family is again applied here to encompass the range in morphology of this cell. The core morphology is conserved, with the SLP, AB and layer 8 of the FB constituting the backbone of this cell type. Several examples of this family are shown in Figure 9 to illustrate both the “familial” characteristics as well as the range of morphologies seen in this group of neurons.

The suggestion that this cell type's morphology is not highly stereotyped, but instead exhibits a conspicuous degree of variability (in other words, that these are all variations of the same cell type), is supported by the observation that the various forms occur in each of the GAL4 and split‐GAL4 lines examined, as well as in both split half parents of a given split‐Gal4 line. For example, a range of morphologies has been seen in VT006486 and two split‐GAL4 lines for which VT006486 is a parent line, SS04457 and SS04423. It is possible that results from behavior and physiology assays, as well as type‐specific split‐GAL4 lines, will indicate these forms are distinct cell types.

Arbors of these family members are predominantly spiny in the SLP and the remaining arbors are composed primarily of boutons and are therefore presumed to be output. The SLP.s‐ABic.b‐FBℓ8.b neuron, the “core” member of the family, is shown in Figures 7c and 9a. The remaining projections in cells in this family seem to exhibit some level of stochasticity, as there is a great deal of variability in the layers of the FB that are targeted. Some cells in this family project neurites that terminate in boutons in various combinations of layers 1, 2, 4, and 5 of the FB, and one bouton was seen in the EB. Typically, just one to several layers receive a bouton or two. The neuron shown in Figure 9b strongly resembles that shown in Figure 9a, but in addition to the relatively robust arbors in the SLP, FBℓ8, and AB, it also extends a few boutons into FB layers 1, 4, and 5 (e.g., red asterisk).

The arbor in FBℓ8 in this family always spans the midline but varies in the degree to which it permeates this layer; as few as five medially located boutons have been seen. The cells shown in Figure 9c,d provide examples of more restricted FBℓ8 arbors. The cells shown in Figure 9b,c are largely similar, except for the extent of the FBℓ8 arbor. Although the neurons in Figure 9b–d have diverse projections, given the presumed stochasticity of these projections we simply refer to them by their family name, SLP‐AB‐FBℓ8.

### 3.6 PB neurons

Since publication of Wolff et al. (2015), several advances have been made in our understanding of neurons that target the PB. First, we have identified several new PB neurons that have features in common with a published cell type, the octopaminergic OA‐AL2i1 cell (Busch, Selcho, Ito, & Tanimoto, 2009). In addition, new insight has been gained into a previously described neuron, PB_G1–8_.s‐EBt.b‐D/V GA.b (Wolff et al., 2015). Finally, a neuron discovered by Lin et al. (PB_1 glomerulus_‐>EB_C_‐IDFB_DSB_ (Lin et al., 2013) has now been identified in GAL4 lines, enabling additional characterization of this cell type, which we call PB_G1‐9_.s‐EBc.b‐D/V GA.b to be consistent with our previously described nomenclature system. The latter two cells, PB_G1‐8_.s‐EBt.b‐D/V GA.b and PB_G1‐9_.s‐EBc.b‐D/V GA.b, share many features in common, as described below. In addition, split‐GAL4 driver lines for the PB neurons have been generated and are presented in Table 2; these lines are generally highly specific for a particular cell type.

#### 3.6.1 A family of PB neurons with morphologically similar PB arbors

**Figure 10:**
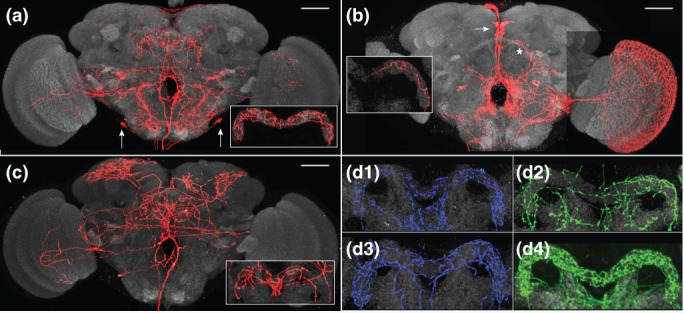
Overview of an octopaminergic‐like family of cells that innervate the PB. The arbors of this neuron family are broadly distributed throughout the brain and resemble previously described octopaminergic neurons (Aso et al., 2014; Busch et al., 2009). Only a subset of arborization patterns is shown in the following images, generated using the MCFO technique. Some of the neurites in these examples do not originate from the octopaminergic neuron innervating the PB cell. The Janelia workstation was used to trace the neurites in three dimensions in confocal images and fragments that were clearly not projections from the cell in question were erased, either in the workstation or using Photoshop. Those neurites that could not be definitively determined to project from other cells were not erased. (a) Most of the arbor in this image appears to belong to the PB octopaminergic cell. The ventral‐most arbors likely emanate from the two cell bodies seen at lower left and right (arrows). Inset: PB (gray) and PB arbor for this cell (extraneous nc82‐positive neuropil was erased using Photoshop in order to highlight the PB.) scale bar = 50 μ [from line VT016610]. (b) This neuron arborizes throughout just half of the PB (asterisk and inset) and most likely projects to the optic lobe. The prominent projection along the dorsal midline (arrow) does not appear to be part of the PB neuron. Scale bar = 50 μ [from line R64F06]. (c) All the neurites seen here appear to be part of the same neuron that innervates the PB. Inset highlights the PB arbor. Scale bar = 50 μ. [from line R12G04]. (d) Additional examples of the range of PB arbors seen in the PB octopaminergic neurons. Neurons shown are from the following lines: (d1) TDC2. (d2) R20F06. (d3) VT027001. (d4) VT004439

We have identified a number of PB neurons that share features in common with one another and with a published PB octopaminergic neuron, the OA‐AL2i1 cell (Busch et al., 2009); see Figure 10 for several examples. Many of these cells have complex morphology, sprouting elaborate networks of projections. In addition, the GAL4 lines in which these cell types were identified show expression in many cells. The combination of the intricate morphology of the cells and crowded GAL4 lines has made it difficult to label individual cells in unique colors, so the descriptions and images that follow are best approximations of the neuronal morphology.

Although all the projections could not be unambiguously traced, there is clearly a wide scope of neuropils targeted by this collection of neurons. Their projections proliferate throughout the brain and some appear to send projections to the optic lobes (Figure 10a–c) and the ventral nerve cord (VNC; not shown). The common feature among these neurons is a sinuous arbor of variable density that courses through the PB (Figure 10d1–d4), suggesting that some PB functions may be modulated by octopamine. In some neurons, the PB arbor traverses just half of the PB (Figure 10b, inset). In other instances, it appears to run the full extent of the PB (Figure 10a, inset; 10c, inset; 10d1–d4), but since there may be two labeled neurons in these specimens the limits of single PB arbors cannot be definitively determined. Since the anatomy of these cells is not well‐defined in our images, we have not named them.

#### 3.6.2 A revised account of the “tile cell,” PB_G1–9_.s‐EBt.b‐D/V GA.b

The functional and anatomical unit of the PB is the glomerulus (G), which derives its name from the Latin “glomus,” meaning “ball of yarn.” In keeping with the standard established in Hanesch et al. (1989) for *Drosophila*, glomerulus is used here rather than the generic term “slice,” which refers to a transverse division of a structure (Ito et al., 2014). The glomeruli are numbered G1–G9, from medial (G1) to lateral (G9). The specific glomeruli targeted by a given cell type are indicated following the neuropil designation PB. The EB is segmented along both its transverse and longitudinal axes. Two distinct transverse volumes are revealed by the arbors that fill them and were termed tiles (abbreviated “EB.t”) and wedges, based on their geometry (Wolff et al., 2015). A third volume, the “canal,” is introduced here.

We identified a cell that bears a strong morphological resemblance to the tile cell, a PB‐EB‐GA cell (Wolff et al., 2015), in seven split‐GAL4 and several generation 1 GAL4 lines. Two significant differences suggested it was a new cell type, but the analysis outlined below indicates they represent the same cell type.

**Figure 11:**
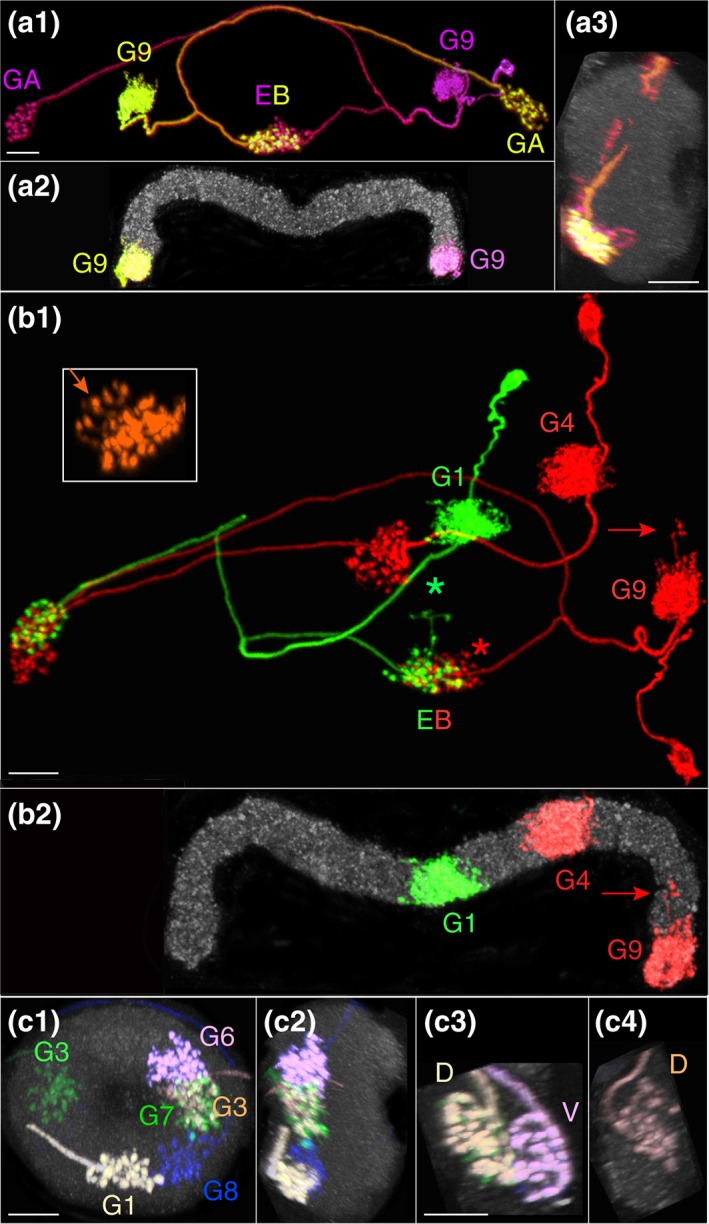
The tile cell: PB_G1‐9_.s‐EBt.b‐D/V GA.b. This cell type was found to target G9 in addition to G1–G8. The new nomenclature reflects this correction. Images obtained using MCFO are shown. (a1) The “new cell” (see text) targets all nine glomeruli of the PB. Two cells that project to G9, where they have spiny arbors, the dorsal gall (GA), and ventral EB tile at 6:00, both filled with varicosities, are shown. Scale bar = 10 μ; also applies to panel a2 [from line SS02191]. (a2) The PB shown here, stained with the α‐nc82 antibody, corresponds to the PB in a1 and is aligned with the glomeruli in a1. This panel illustrates the lateral location of the neurons in the PB. (a3) The yellow and pink EB arbors from the G9 cells shown in (a1) and (a2) are confined to the posterior shell, seen in this sagittal view. Anterior is to the right. Scale bar = 10 μ. (b1) MCFO image showing three tile cells innervating G1, G4, and G9. The tile cells that target G1 and G9 occupy the same domain in the EB, although their arbors are slightly offset on opposite sides of the midline. This G1 cell extends tendrils from its EB arbor (green asterisk) whereas its red neighbor does not. See b2 legend for feature highlighted by red arrow [from line SS02191]. Inset shows an EB arbor with the largest number of tendrils seen to date in a tile cell. Scale bar = 10 μ; also applies to panel b2 [from line SS02191]. (b2) α‐nc82 label is included as a reference to illustrate the glomeruli targeted by the PB_G1‐9_.s‐EBt.b‐D/VGA.b cells shown in (b1). The PB in (b2) is aligned with the PB in (b1). PB arbors sometimes extend into neighboring glomeruli (red arrow in b1 and b2). (c1) The odd/even glomerulus:gall rule is obeyed by the tile cell. The projections of three cells are followed from the EB to the gall in (c). Input for these cells comes from PB G1 (yellow), G3 (orange), and G6 (pink). Their EB tile domain arbors are shown in frontal (c1) and sagittal (c2) views. The EB arbors of the cells that project to G7 (green) and G3 (orange) of the PB occupy the same tile domain in the EB. Scale bar = 10 μ; also applies to panel c2 [from line SS27853]. (c2) Sagittal view of the EB shown in (c1) illustrates the depth of the EB arbors is confined to the posterior shell, which occupies the posterior third of the EB. Anterior is to the right. (c3) Projections of the G1 (yellow) and G6 (pink) cells in c1 to their respective gall domains. Cells that target the odd glomeruli, G1 (yellow) and G3 (orange, panel c4), project to the dorsal gall domain (D), whereas the cell that targets the even‐numbered glomerulus, G6 (pink), projects to the ventral gall domain (V). Images are rotated to reveal that these arbors fill their respective compartments. D = dorsal gall. V = ventral gall. Scale bar = 10 μ; also applies to (c4). (c4) Same as described for c3, but in this case, the dorsal gall is arborized by the PB cell that targets G3 (orange)

We originally reported that the tile cell targets only eight of the nine glomeruli, skipping the most lateral glomerulus, G9, and that its EB arbor, characterized in GAL4 line R33A12, is confined to the posterior ring of the EB. In contrast, the apparently new cell targets all nine glomeruli (Figures 11a1,a2,b1,b2 and 12a1,a2). Furthermore, a small subset of the new cell's EB arbors project tendrils that reach toward the central canal of the EB (Figure 11b1, green asterisk; compare to neighboring red arbor that lacks these tendrils, red asterisk). Each of these filamentules terminates in a single bouton (Figure 11b1, inset, arrow). The number of bouton‐capped tendrils varies from cell to cell, from zero, as was seen with the original tile cell in line R33A12, to several (Figure 11b1 inset).

In other respects, these two cells are indistinguishable. First, their PB arbors are both spiny and fill their resident glomeruli, often spilling into a neighboring glomerulus (Figure 11b2, red arrow). Second, both EB arbors are confined to the posterior shell (Figures 11a3,c2 and 12a3 right). And third, the GA arbors consist primarily of boutons, which fill either the dorsal or ventral subcompartment of the GA, depending on the glomerulus of origin (Figures 11c3,c4 and 12a4; cells that arborize in the odd glomeruli navigate to the dorsal gall and those that target even glomeruli track to the ventral gall, as described in Wolff et al. (Wolff et al., 2015).

**Figure 12:**
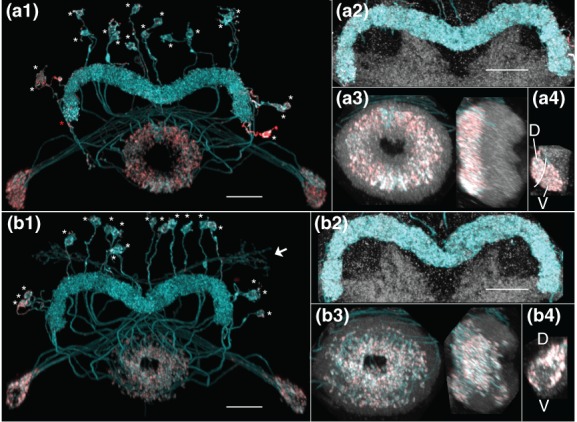
Population‐wide view of tile and canal cells. (a1) SS02191‐driven expression of a membrane‐targeted epitope (blue) and a presynaptically‐targeted epitope (red). Line SS02191 shows expression in only the tile cell: PB_G1‐9_.S‐EBt.B‐D/V GA.B. Asterisks identify cell bodies; red asterisk identifies primary neurite that is missing its cell body, which are sometimes lost in dissection (red syt signal seen in the cell bodies is likely due to protein trapped in the golgi, as is frequently seen with exogenously expressed proteins). Scale bar = 20 μ. (a2) Cells in PB are labeled with a membrane marker (blue) and reference channel is identified with α‐nc82 label (gray). All 18 glomeruli of the PB are targeted by this cell type. Scale bar = 20 μ; also applies to a3 and a4. (a3) The radius (left) and depth (right; sagittal view of EB) of the EB arbor are shown; the reference channel is included to identify the EB boundary. The arbor does not extend to the canal and populates only the posterior third of the EB (anterior is to the right in sagittal view). (a4) Both compartments of the gall are densely packed with boutons. White line delineates the boundary between the dorsal and ventral gall compartments. α‐nc82 label to the right of the gall is not part of the gall. (b1) SS02195‐driven expression of the canal cell: PB_G1‐9_.S‐EBc.B‐D/V GA.B. Markers are the same as described in Figure 12a1 legend. Cell bodies were identified in the confocal stack and their locations are indicated by asterisks in this maximum intensity projection, where they are difficult to resolve. Faintly stained ramifications belong to an unrelated cell type (arrow). Scale bar = 20 μ. (b2) The canal cell also populates all 18 glomeruli of the PB. Cells are labeled with a membrane marker, PB labeled with α‐nc82. Scale bar = 20 μ; also applies to (b3) and (b4). (b3) The canal cell EB arbor is offset toward the canal (left) and extends the depth of the EB (right, sagittal view, anterior is to the right). (b4) Also in contrast to the tile cell, the canal cell gall arbor does not fill the dorsal and ventral domains. The ventral gall arbor clearly wraps around the ventral gall. Refer to MCFO images in Figure 13 to better visualize the distribution of the dorsal gall arbor

**Figure 13:**
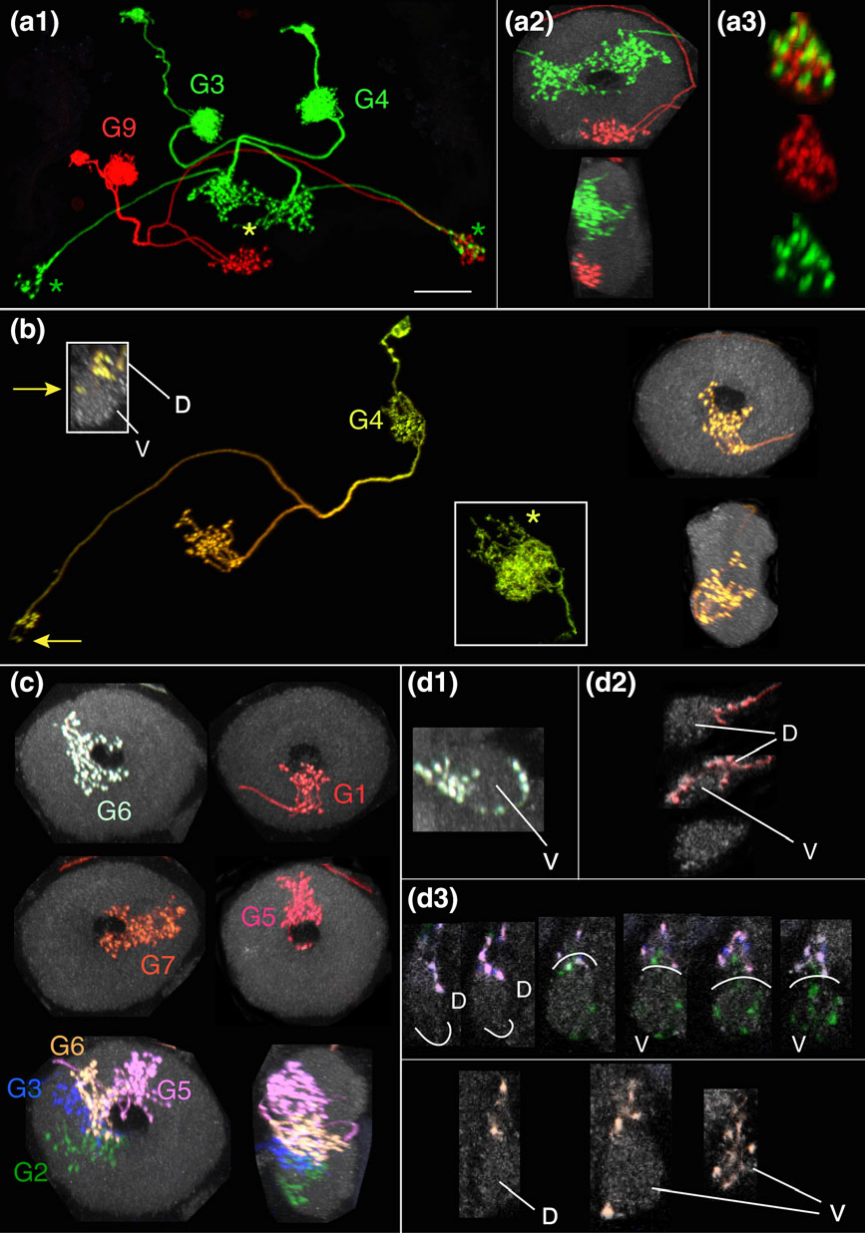
The canal cell: PB_G1‐9_.s‐EBc.b‐D/V GA.b. (a1) Line SS02198 shows expression in both the canal and tile cells and enables a direct comparison between the two cell types. Shoots from the EB arbors of the two green canal cells enwrap the EB canal (yellow asterisk; see also a2) whereas such filamentules are notably absent from this red tile cell (and most tile cells). Note the distinct difference in density of the gall arbors: the red tile cell arbor has more boutons than the sparse canal arbors (green asterisks). G3, G4, and G9 identify the glomeruli of the PB that are targeted by these cells. Scale bar = 20 μ [from line SS02198]. (a2) Footprints of the tile (red) and canal (green) cells differ in radius (top, frontal view) and depth (bottom, sagittal view). Both cell types occupy the posterior shell of the EB; the canal cell projects even deeper, into the medial and sometimes even the anterior shells, although only a few boutons reach the anterior shell. Anterior is to the right in bottom panel. (a3) Canal cell gall arbors (green) are sparser than tile cell gall arbors (red) and generally even less dense than in this sample, for example, as seen in Figure 13b,d. (b) A canal cell that targets G4 in the PB. As noted for the tile cell, PB arbors from canal cells can also spill into neighboring glomeruli, shown in the right inset, in which G9's arbor extends fine, sparse shoots into G8 (asterisk). The canal cell was named for the filamentules that project from the EB arbor toward the central canal (top right). Sagittal view of the EB (lower right) illustrates that the bulk of the canal arbor is concentrated at the posterior of the EB, with a few branches reaching more anteriorly (anterior is to the right). Note that this cell, which targets an even‐numbered PB glomerulus, projects to just the tip of the ventral gall and sends a small projection more ventrally (arrows). The canal cell gall arbor is not as robust as the tile cell's gall arbor in that it does not fill the gall. The dorsal gall is in a different plane of view than is shown in the inset and does not receive input from this cell [from line SS02195]. (c) The distribution of the canal cell's EB arbor is variable; in some cells, boutons almost completely enwrap the canal (G5 and G6, in this figure), in others, a uniform swath extends the radius of the EB (G7), and in still others, the greatest density of boutons is offset toward the canal (G1). Several additional examples and the depth to which they penetrate the EB are shown in the lower panel; only the orange cell (G6) reaches the anterior shell, as seen in the side view (anterior is to the right). The projections of these cells to the dorsal/ventral gall are shown in (d) [SS02195, SS02198, SS04776]. (d) The profile of the canal cell's gall arbor is distinct from that of the tile cell but like the tile cell, it follows the odd/even glomerulus to D/V gall projection rule. D = dorsal gall. V = ventral gall. (d1) The mint green G6 cell from (c) projects to the ventral gall (V) in panel d1 but rather than filling this neuropil, as does the tile cell (e.g., red cell in Figure 13a3), it appears to track along this neuropil's surface [SS02198]. (d2) The red G1 cell shown in (c) also exhibits this tracking behavior in the gall. This three‐panel series from posterior (top panel) to anterior illustrates that this red arbor snakes between the dorsal (D) and ventral (V) gall compartments. Note the main body of the DGA (D) is void of boutons. Instead, the varicosities coat the surface of the DGA that juxtaposes the VGA (V; note the VGA peeking through as the DGA gives way to the underlying VGA in the middle panel). There are no boutons within or on the surface of the VGA [SS02195]. (d3) These gall arbors correspond to the cells shown in (c). Again, the two series shown run from posterior (left) to anterior. Upper panel, the pink cell (originates from PB G5) targets the dorsal gall (D), the green cell (originates from G2) targets the ventral gall (V). Lower panel, the orange cell (originates from G6) targets the ventral gall (V). The GA arbors appear to mostly “surround” the gall compartments more than fill them. This is particularly evident in the right panels of the lower series, in which the ventral gall‐seeking cell does not fill the VGA (gray neuropil) but instead tracks along its anterior surface. The white lines delineate boundary between dorsal and ventral gall compartments. Scale bar = 20 μ [SS04776]

Two approaches were taken to determine if the variants represent the same cell type. First, additional R33A12 MCFO brains were examined for cells with tendrils and cells that target G9. Of 66 MCFO‐labeled PB_G1‐9_.s‐EBt.b‐D/V GA.b cells in line R33A12, just one exhibits a single spindle projection terminating in a bouton and only three cells project from G9, a lower than statistically expected frequency. The presence of this cell type in G9, combined with the strong morphological resemblance of the two variants, suggests the variants are the same cell type.

Second, flies from hemidriver lines representing each variant were crossed and the progeny examined for cells that both arborize in G9 and elaborate tendrils.

AD and DBD split half parents from the tile cell line, R33A12, were crossed to complementary split half parents that drive expression in a “tile‐like” cell in line VT040589. The tile cells present in the progeny of this cross (line SS27853; Figure 11c) frequently target G9 (not shown), confirming the two variants are not only one and the same type, but also that this class of neurons arborizes in all nine glomeruli. Furthermore, the three SS lines that include VT040589 as a split half parent (SS02191, SS27853, and SS16814), all drive expression in the EB tile cell in all nine glomeruli (e.g., SS27853, Figure 11). This cell's name is therefore being changed from PB_G1‐8_.s‐EBt.b‐D/V GA.b to PB_G1‐9_.s‐EBt.b‐D/V GA.b to reflect its presence in G9 (note the change in the name is from G1‐G8 to G1‐G9).

The 1:1 projection pattern between G1 and G8 in the PB and corresponding domains in the EB and GA for the tile cell is described in detail in Wolff et al. (2015, Figure 14b). Briefly, cells that arborize in G1R/G1L project to the ventral tile of the EB, at approximately 6:00 on a clock face; cells in G2R/G8L project to 7:00–8:00, G3R/G7L project to 8:00–10:00, G4R/G6L to 10:00–11:00, G5R/G5L to 11:00–1:00, G6R/G4L to 1:00–2:00, G7R/G3L to 2:00–4:00, and cells in G8R/G2L project to 4:00–5:00. Cells that target G9 were not previously characterized. Analysis of the G9 subset of tile cells in GAL4 and SS lines revealed they project to the same EB tile as G1R and G1L, so the 6:00 tile receives input from the medial‐most and lateral‐most glomeruli of the PB (Figure 11a1,a2,b1,b2). These data also show that tile cells that target G9 strictly adhere to the previously described PB glomerulus:GA wiring pattern, so cells that arborize in G9 target the dorsal gall (not shown).

#### 3.6.3 The canal cell, PB_G1‐9_.s‐EBc.b‐D/V GA.b, strongly resembles the tile cell

Since publication of Wolff et al. (2015), MCFO performed on additional GAL4 lines revealed a PB‐EB‐GA cell not reported in that study. Also in Wolff et al. (2015), an effort was made to correlate the PB cell types described in that report with PB cell types described in Hanesch et al. (1989) and Lin et al. (2013); see Table 3A,B in Wolff et al. (2015). At the time, the closest match to the tile cell (PB_G1‐9_.s‐EBt.b‐D/V GA.b) was PB_1 glomerulus_‐ > EB_C_‐IDFB_DSB_ from the Lin et al. (2013) paper. We now believe the “canal cell,” not the tile cell, is equivalent to the PB_1 glomerulus_‐ > EB_C_‐IDFB_DSB_ cell. (Currently, there is therefore no Lin et al., 2013 correlate for the tile cell.) Since MCFO enables a detailed analysis of circuitry and morphology, here we build upon the description provided in Lin et al. (2013) and compare the canal cell to the morphologically similar tile cell.

The canal cell's name is derived from a prominent anatomical feature of this neuron: its EB arbor partially enwraps the canal, or central hole, of the ellipse (Figure 13). Following the nomenclature system adopted in Wolff et al. (2015), this cell's formal name is the PB_G1‐9_.s‐EBc.b‐D/V GA.b cell, where the “c” that follows “EB” stands for canal. The canal and tile cells share many features in common, including overall morphology, putative input and output regions, and circuitry. First, light‐level analysis reveals that the canal cell has primarily boutons in the EB and gall (“.b” for boutons; Figure 13a–d) and predominantly spiny arbors in the PB (“.s” designates a spiny morphology; Figure 13b, right inset), as described for the tile cell. Furthermore, the PB arbors for both cells often occupy more than one glomerulus, spilling over into the neighboring glomerulus (Figure 13b, right inset, asterisk). Occasionally, the arbor completely fills two neighboring glomeruli and a small portion of a third (not shown). Second, the canal cell also targets all nine glomeruli of the PB (Figure 12b1,b2). Third, canal cells also respect the odd/even glomerulus:D/V GA wiring pattern (Figure 13c,d for several examples). Fourth, the circuitry between individual glomeruli and the corresponding regions in the EB is identical between the two cell types [not shown; see Figure 14b from (Wolff et al., 2015) for visual depiction of this circuitry].

The key differences between the tile and canal cells are the distribution and morphology of the EB and GA arbors. The canal cell EB arbor projects shoots of variable length around a portion of the circumference of the canal, partially enwrapping the canal (Figure 13a1,a2,b,c). Relative to the tile cell, the bulk of the canal cell EB arbor is shifted from the periphery to the center. The density of boutons in the canal EB arbor and the area over which the arbor is dispersed from periphery to center varies significantly from cell to cell (Figure 13a1,a2, green arbors; 13b, top right: 13c). Consequently, in some instances it is difficult to distinguish the tile and canal cells from one another.

The depth of the EB arbor also differs between the two cell types. Whereas the tile cell EB arbor is confined to the posterior ring (Figure 12a3, right), the canal cell EB arbor occupies not only the posterior ring, but also invades the medial and sometimes even the anterior ring of the EB (sagittal views in Figures 12b3 and 13a2,b,c).

The gall arbors in the tile and canal cells are usually distinct, although sometimes they, too, are hard to differentiate. The tile cell's gall arbor is robust and fills the entire volume of the dorsal or ventral gall (Figures 11c3,c4 and 12a4). In contrast, the canal cell's gall arbor is much sparser (Figures 12b4 and 13a1, green asterisks; 13a3, green arbor; 13b, left inset and arrows; 13d) and appears to either creep along the surface of the gall or snake between the dorsal and ventral compartments of the gall (Figure 13d1,d2). Sometimes it seems to hover at the dorsal or ventral tip (Figure 13d3, pink cell).

While the variability in morphology can make it difficult to distinguish between the tile and canal cells at the single cell level, distinctions between the two cell types are unmistakable when the entire population of each cell type is labeled in split‐GAL4 lines specific for each cell type (compare tile cell in Figure 12a to canal cell in Figure 12b). These images confirm the MCFO‐based observations noted above, as follows. First, all nine glomeruli are targeted by both cells (Figure 12a1,a2,b1,b2). Second, the tile cell EB arbor does not extend to the canal (Figure 12a3, left), it fills only the posterior shell of the EB (Figure 12a3 right; anterior to the right), and the arbors in the dorsal and ventral gall are dense and uniform (Figure 12a4). In contrast, the canal cell's EB arbor exhibits a significant concentration around the canal (Figure 12b1,b3 left) and is dispersed throughout the posterior, medial, and anterior rings of the EB (Figure 12b3, right), and the gall boutons are not densely concentrated throughout this neuropil, instead appearing to be concentrated dorsal to the dorsal gall and around the periphery of the ventral gall (Figure 12b4).

#### 3.6.4 A renamed PB cell

The PB_G1‐8_.s‐FBℓ3,4,5.s.b‐rub.b cell is being renamed PB_G1‐8_.s‐FBℓ3,4,5.s.b‐ROB.b. We misidentified the rubus (RUB) in the original classification; instead, this arbor targets a structure named the round body by Lin et al. (2013). While this structure is abbreviated “RB” by Lin et al., K. Shinomiya proposes ROB to avoid confusing it with RUB (personal communication).

## 4 DISCUSSION

There is increasing evidence that many complex insect behaviors, such as navigation and sleep, rely crucially on the central complex, yet our understanding of this key brain region is in its infancy—even the fundamental building blocks of its neuropils are, for the most part, uncharacterized. With the current tools available in *Drosophila*, comprehensively cataloging these neurons and generating the tools to manipulate them is a logical and feasible first step, the results of which will facilitate a more sophisticated understanding of the circuit computations and the behaviors controlled by neurons of the central complex. With a catalog of neurons, a set of genetic driver lines that target each of these cell types and a battery of behavioral assays through which to run these targeted fly lines, the tools exist to quantify the behavioral effects of manipulating the activity of defined, small subsets of neurons. Such experiments provide a way to infer the involvement of different neuron types and neuronal networks in specific behaviors. The work described here provides such a descriptive catalog of neurons and genetically targeted GAL4 driver lines for two structures, the noduli and the asymmetrical body, as well as GAL4 driver lines for PB neurons. The availability of these tools will facilitate studies of the roles of the NO, AB, and PB and the neuronal cell types that populate these brain structures.

### 4.1 The asymmetrical body: The fifth central complex structure

The neuropils considered to be constituent components of the central complex have changed over the decades. Power (1943) defined the neuropils of the central complex to include the “central body, the ellipsoid body and the pair of ventral tubercles.” The central body (Flögel, 1878), included what became known as the ellipsoid body (Lowne, 1892) and the fan‐shaped body (Dietl, 1876); reviewed in (Strausfeld & Seyfarth, 2008). The noduli were known as the ventral tubercles. More recently, the central complex has been defined as “a group of modular neuropils across the midline of the insect brain” (Pfeiffer & Homberg, 2014), “…interconnected neuropils and nuclei that populate the midline of the forebrain‐midbrain boundary region” (Strausfeld & Hirth, 2013), and “a system of interconnected neuropils lying at, or about, the midline of the protocerebrum” (Ito et al., 2014). Although the modular architecture of the central complex structures is conspicuous (e.g., the glomeruli of the PB and the trajectory patterns of neurons that project to, from, and within the central complex structures), it is the assigned boundaries that encompass the central complex that seem to be the feature that defines these structures as members of the central complex.

Here, we illustrate that the *Drosophila* AB, which appears to be a structure that is distinct from the FB, meets the criteria outlined above for central complex neuropils: It is a midline neuropil; it falls within the boundaries defined by Power (1943) and Strausfeld and Hirth (2013); and it is interconnected (to the FB and SLP) by a network of previously undocumented (with one exception) neurons. Since the AB meets all the criteria previously used to define neuropils as components of the central complex, we propose that the AB be added as a fifth neuropil of the central complex of *Drosophila*.

The AB is not unique to *Drosophila*. Phillips‐Portillo and Strausfeld (2012) describe the presence of likely homologous bilateral, asymmetrically sized ABs in *N. bullata* and *C. erythrocephala*. Their work also identifies a tangential FB neuron that bears a resemblance to the SLP‐AB‐FBℓ8 neuron described here. It remains to be determined if the AB is more widely represented in other insect orders.

### 4.2 A mostly asymmetric neuron

The right AB is significantly larger than the left. At a minimum, this difference is likely due to a combination of smaller arbors in the left AB and the lower frequency with which the left AB is targeted: only the ipsilateral‐contralateral‐projecting form of the SLP‐AB neuron, which arborizes in both the left and right ABs, targets the left AB, whereas the ipsilateral and contralateral‐projecting forms of the SLP‐AB neuron target exclusively the right AB. Thus, the right AB appears to receive a disproportionately larger share of information from the SLP, although the right and left hemispheres appear to be equally represented as sources of input. Notably, this left–right bias is restricted to the AB, as a parallel preference is not shown for the SLP. The availability of genetic lines that target AB‐specific cell types will enable experiments aimed at revealing the relevance of this left–right bias.

### 4.3 Unusual properties of the NO neurons

Three unusual features distinguish the five most commonly seen NO neurons described here from other central complex neurons. First, in contrast to the majority of PB neurons described to date, the projections of four of these five NO neurons are ipsilateral. Second, while anatomical features identify distinct input and output neuronal populations in other central complex neuropils, the noduli appear to be sites for receiving primarily input from other neuropils (boutons appear to be the predominant anatomical feature in the noduli in confocal micrographs of NO, PB‐FB‐NO, and PB‐EB‐NO neurons). Golgi preparations and data from the likely locust equivalent of the PB‐FB‐NO neuron (the CPU4 neuron), however, indicate these NO arbors are mixed (Hanesch et al., 1989; Heinze & Homberg, 2008; Stone et al., 2017); perhaps the intensity of the dense populations of boutons masks the presence of spines in confocal preparations. Third, although the noduli do receive input from central complex neuropils (e.g., via the PB‐FB‐NO and PB‐EB‐NO neurons, from FB tangential neurons, etc.), the majority of direct input for this new set of neurons is provided by just one neuropil, the LAL. Such a restricted thoroughfare of communication is in stark contrast to the PB neurons, for example, which have a much broader and more diverse network of direct communication.

The LAL.s‐CREc.s‐NO_3_Pc.b cell type is distinct from the other four common NO neurons in that it delivers contralateral, rather than ipsilateral, input from the LAL and CRE to NO_3_P. The posterior compartment of NO_3_ is therefore unique in that it is the only nodulus subcompartment to communicate directly with the contralateral hemisphere. Given that NO_3_P (and NO_3_M) also receives ipsilateral terminals from the LAL via LAL.s‐CREi.s‐NO_3_P/Mi.b, this subcompartment may act as a limited integration center between the fly's left and right sensory fields.

### 4.4 Potential roles of the NO neurons

Physiological data from two neurons in the sweat bee offer insight into a likely role for the NO neurons described here. The TN1 and TN2 neurons (“noduli tangential neurons”) share a high degree of anatomical homology with the LAL‐NO neurons: TN1 and TN2 are ipsilateral neurons with input branches in the lateral central brain and blebbed branches in the noduli (Stone et al., 2017). Recordings from these two cells reveal they fire in response to simulated backward and forward flight, respectively, and that the rate of firing is dependent on the stimulus velocity, suggesting these neurons encode speed using optic‐flow and can thereby track the distance traveled by the bee. Similar physiological features and path integration functions would not be unexpected for the apparent homologous *Drosophila* neurons.

The LAL is the primary source of input for the NO neurons described here and its activity may provide additional insight into the roles of the LAL‐NO neurons. It is a large, bilateral neuropil that is highly interconnected with neuropils of the central complex. Functionally, the LAL is considered to be a sensorimotor integration center, based on several lines of evidence in various insect species. For example, in crickets and moths, activity in LAL neurons is associated with walking (Iwano et al., 2010; Mishima, 1999; Zorovic & Hedwig, 2013). In the locust, assorted LAL neurons exhibit changes in activity in response to various aspects of flight, implicating this brain region in flight control (Homberg, 1994). In *Drosophila*, LAL neurons involved in walking backwards have been documented (Bidaye, Machacek, Wu, & Dickson, 2014).

It has been suggested that the noduli are involved in walking and motor control in *Drosophila* (Buchanan et al., 2015; Strauss & Heisenberg, 1993). The neurons implicated in left–right turning bias in locomotion are the PB‐FB‐NO neurons (Buchanan et al., 2015), which have presumed input (fine terminals) in the PB, and presumed output (boutons) in the FB and NO. The authors speculate that the bias to turn in one direction or the other is influenced by an interplay between the nodulus subdomains that are targeted by the different PB‐FB‐NO cell types. Direct communication between the PB‐FB‐NO neurons and the LAL‐NO neurons is not unexpected, as Stone et al. (2017) have shown synaptic contacts between the bumblebee equivalents of these two cells, the CPU4 and TN cells, respectively. Considering the sensorimotor contribution made by the LAL in various types of movement, the LAL‐NO neurons described here are strong candidates to contribute to the circuits involved in turning.

### 4.5 NO neurons: An incomplete picture

The catalog of NO neurons described here is incomplete. Our analyses of other GAL4 lines have identified several large‐field FB neurons that also arborize in the noduli, as well as other brain regions that we are currently characterizing; some of these neurons are illustrated in the Golgi stains of Hanesch et al. (1989). Hanesch et al. (1989) also describe other cell types with arbors in the noduli that have so far eluded our identification. Finally, it seems likely that there would be output neurons from the NO, although we have not yet identified such neurons in our studies. Electron microscopic‐level analysis should provide a path to identifying these neurons.

### 4.6 Defining “cell type” and neuropil boundaries

The debate continues to swirl over what constitutes a distinct cell type. Morphology and function have long been accepted as reliable criteria to distinguish cell types. While morphology is a straightforward and easy means of classifying cell types, it can be misleading in that cells that appear identical may have functional differences. For example, Green et al. (2017) describe clearly distinct physiological roles for two PB neurons that appear to have indistinguishable morphology at the light level. Morphological features evident with light microscope‐level resolution will therefore likely be insufficient to distinguish all cell types, so knowledge of some combination of synaptic connectivity, functional properties and the genetic programs used to specify these attributes will be necessary to fully define cell types.

Similar limitations confound the assignment of neuropil boundaries and subcompartments. Synaptic density varies considerably across brain regions and this variation has provided landmarks used to define the neuropils of the fly brain (Ito et al., 2014). While the boundaries of some structures are unambiguous (e.g., the PB and EB), neuropil margins are not universally so clear‐cut, with many neuropils appearing to meld seamlessly with adjacent neuropils. The opportunity to map the domains of arbors within neuropils identifies distinct regions that are not revealed by differences in synaptic density (e.g., wedge and tile domains in the EB). For example, the mushroom body lobes can be divided into a series of nonoverlapping compartments with distinct functions by the extent of the arbors of dopaminergic input neurons and mushroom body output neurons (Aso, Hattori, et al., 2014; Aso, Sitaraman, et al., 2014). The LAL provides an example of one neuropil that may have functionally distinct subregions. It is a large neuropil with no obvious boundaries revealed by anti‐Brp staining, yet the arbors of many neurons that target this neuropil exhibit strong regional preferences. Mapping the domains of these arbors may identify regions that are functionally distinct.

### 4.7 Obtaining a comprehensive description of cell types, circuits, and the tools to manipulate them

Three major efforts aimed at cataloging all the neurons in the *Drosophila* brain are in progress. One, typified by this and others' work (Aso, Sitaraman, et al., 2014; Tuthill, Nern, Holtz, Rubin, & Reiser, 2013; Wu et al., 2016), characterizes one structure at a time using light microscopy in combination with the generation and analysis of highly specific GAL4 driver lines. The second method is a modern implementation of the Golgi approach of randomly labeling small numbers of neurons in order to describe their morphology (Chiang et al., 2011). And the third, which is now becoming practical at the required scale, involves reconstruction of neuronal morphology and circuits through analysis of image volumes collected using electron microscopy (see, for example, Takemura et al., 2017, Takemura et al., 2013). We believe that such light and electron microscopic‐level analyses will be highly synergistic. Light microscopy, with genetically marked cells, provides the ability to observe the morphology of hundreds of individual cells of the same cell type in many different individuals, providing insights on stereotypy. However, its dependence on GAL4 drivers means that completeness of coverage cannot be assured. Conversely, electron microscopic analysis, while usually limited to a single sample, not only ensures completeness but also enables visualization and quantification of synaptic connectivity. Moreover, since EM samples do not carry transgenes expressing ectopic membrane proteins that can interfere with development, wiring errors may be less likely. While only electron microscopy is likely to provide the complete wiring diagram of a circuit, light level analysis of genetic driver lines will be needed to provide the critical bridge between circuit maps and the tools required to precisely manipulate the activity of their individual components.

## 5 ACKNOWLEDGMENTS

The authors are grateful to Laszlo Tirian and Barry Dickson for providing *Drosophila* lines (the “Vienna collection”) prior to publication and to Jens Goldammer, Masayoshi Ito, Ryo Minegishi, and Aljoscha Nern for generously sharing MCFO data. We thank A. Nern for data that was used to generate panels in Figure 10. The first iteration of the rules for neuron abbreviations described here came from a brainstorming session that included Ben de Bivort, Jonathan Green, Stanley Heinze, Vivek Jayaraman, Kyobi Kakaria, Gaby Maimon, and Tanya Wolff. An anonymous reviewer proposed the use of subscripts in the abbreviations. The authors thank Hideo Otsuna, J. Goldammer, and Yoshi Aso for assistance with FluoRender; H. Otsuna for running mask searches; H. Otsuna and Mike Dolan for writing scripts that facilitated data processing; and M. Dolan for creating the paired dot and box plots. We thank Arnim Jenett for several of the stable split lines reported here. Dan Turner‐Evans generously provided unpublished insight into the identity of the P‐EN cells in line SS04912. Crystal diPietro provided help with table formatting. This work would not have been possible without the dedicated efforts of the Janelia Fly Core, who sets up crosses, members of Janelia Fly Light, who do the dissections and immunohistochemistry, and members of the imaging team, who image the fly brains. Scientific Computing supports the Janelia Workstation, which is used to view the confocal stacks. We thank V. Jayaraman for insightful discussions and Kazunori Shinomiya for his input on the ROB abbreviation. We are grateful to Chris Doe, Ulrike Heberlein, V. Jayaraman, Nick Strausfeld, and two anonymous reviewers for critical and valuable comments on the manuscript.
